# Food-Associated Kounis Syndrome: Clinical Spectrum and Diagnostic Considerations from a Case Report and Systematic Qualitative Synthesis of Published Cases

**DOI:** 10.1007/s12016-026-09210-1

**Published:** 2026-10-11

**Authors:** Sergio Sanchez-Fernandez, Gabriel Gastaminza Lasarte, Carmen D’Amelio Garófalo, Enrique Paredes-Gutierrez, Alfonso Calvo Alonso, José Bodero-Sánchez, Edurne Cerrada Uribesalgo, Lucía Parrado Gil, Irene Martín De Prado, Jimena Crespo Quirós, Laura Argiz Álvarez, Aitor Hernández Hernández, Elisa Fernández Elías, Teresa Toscano de las Heras

**Affiliations:** 1 Clínica Universidad de Navarra, Pamplona, Spain; 2 Clínica Universidad de Navarra, Madrid, Spain; 3 Fundación Hospital de Calahorra, Calahorra, Spain; 4 Tecnológico de Monterrey, Monterrey, Mexico

**Keywords:** Kounis syndrome, Food allergy, Anaphylaxis, Acute coronary syndrome, Coronary vasospasm, Mast-cell activation

## Abstract

Kounis syndrome (KS) is a hypersensitivity-associated acute coronary syndrome. Food-associated KS remains poorly characterized, and its clinical presentation may be mimicked by non-immune food-related toxic reactions. To characterize the food exposures, clinical and cardiovascular presentations, allergological assessment, potential cofactors, KS phenotypes, management, and outcomes reported in food-associated KS. PubMed, Scopus, and Web of Science Core Collection were searched from inception to August 22, 2026. Original case reports, case series, and sufficiently informative conference reports describing objective coronary involvement temporally associated with a food or food-derived exposure were included. Patient-level data were synthesized qualitatively. KS diagnostic certainty, coronary phenotype, food causality, and presumed mechanism were assessed separately. Planned sensitivity analyses restricted the synthesis to confirmed or suspected food allergy, definite or probable KS, and cases without epinephrine administration before coronary onset or its first objective documentation. The synthesis included 50 publications or reports describing 53 unique patients. Food-related mechanisms comprised confirmed food allergy in 20 patients, clinically suspected food allergy in 26, scombroid poisoning in six, and a Coprinopsis atramentaria-alcohol interaction in one. The reported coronary spectrum ranged from transient vasospasm and nonobstructive presentations to plaque-related myocardial infarction, native-coronary thrombosis, and stent or graft thrombosis. Type I was the most frequently assigned phenotype. Allergological assessment, paired acute and baseline tryptase measurements, treatment chronology, and long-term follow-up were inconsistently reported. The main cardiovascular patterns remained broadly consistent across the planned sensitivity analyses. Food-associated KS encompasses a heterogeneous spectrum of allergic coronary presentations. Clinically similar non-immune food-related toxic reactions, including scombroid poisoning, should be recognized as differential diagnoses and analyzed separately from true allergic KS. Diagnosis requires integration of exposure chronology, objective cardiovascular findings, coronary substrate, allergological evidence, potential cofactors, and competing exposures. Intramuscular epinephrine remains the first-line treatment for anaphylaxis, and temporal precedence should not be interpreted as evidence that epinephrine caused the coronary event.

**Supplementary Information:**

The online version contains supplementary material available at https://doi.org/10.1007/s12016-026-09210-1.

## Introduction

 Kounis syndrome (KS) is a hypersensitivity-associated acute coronary syndrome characterized by the concurrence of an acute hypersensitivity reaction and coronary involvement, which may manifest as coronary vasospasm, myocardial infarction, native-coronary thrombosis, or stent thrombosis [1]. First described as allergic angina by Kounis and Zavras in 1991, KS reflects a complex interaction between immune activation, platelet responses, and the underlying coronary substrate [2]. Mast-cell activation is central to its proposed pathophysiology. In IgE-mediated reactions, allergen-induced cross-linking of specific IgE bound to high-affinity FcεRI receptors activates mast cells and basophils, leading to the release of vasoactive, inflammatory, and prothrombotic mediators [3]. These include histamine, platelet-activating factor, tryptase, chymase, leukotrienes, thromboxane, cytokines, and chemokines [1, 3, 4]. Histamine, leukotrienes, and thromboxane may promote coronary vasoconstriction and endothelial dysfunction, whereas tryptase and chymase may contribute to atherosclerotic plaque destabilization through activation of matrix metalloproteinases. Platelet-activating factor and thromboxane may additionally promote platelet activation, aggregation, and thrombosis [1, 4, 5]. However, KS may also occur through non-IgE-mediated pathways. The resulting coronary phenotype therefore depends on both the characteristics of the hypersensitivity response and the patient’s underlying coronary anatomy. Vasospasm may predominate in patients without established coronary disease, whereas plaque erosion, plaque rupture, or thrombotic occlusion may occur in patients with underlying atherosclerosis, and stent thrombosis may develop in patients with intracoronary devices [1, 4, 5]. Three principal KS variants are conventionally recognized. Type I occurs in patients without established coronary artery disease and is characterized by coronary vasospasm, with or without myocardial injury and elevation of cardiac biomarkers. Type II occurs in patients with underlying atherosclerotic disease, in whom the hypersensitivity reaction may induce vasospasm or precipitate plaque destabilization and acute myocardial infarction. Type III comprises hypersensitivity-associated stent thrombosis or, less commonly, in-stent restenosis, and mast cells or eosinophils may be identified in aspirated thrombotic material [1, 4, 6]. A fourth variant has been proposed to describe hypersensitivity-associated thrombosis or vasospasm involving coronary artery bypass grafts, although its clinical boundaries and frequency remain insufficiently established [5]. The relative frequency of these phenotypes remains uncertain because the available evidence consists predominantly of case reports, small case series, and retrospective observational studies [1, 7, 8].

Although drugs are among the most frequently reported triggers, KS has also been associated with foods, Hymenoptera stings, contrast media, environmental exposures, and materials used in coronary devices [1, 5, 7, 8]. Food-associated KS remains comparatively undercharacterized and presents specific difficulties in causal attribution. In addition to immune-mediated food allergy, food-associated coronary events may occur in the context of non-immune mechanisms, including scombroid poisoning caused by exogenous histamine and other food-related toxic reactions. These mechanisms should not be classified indiscriminately as IgE-mediated food allergy, even when their clinical manifestations resemble an allergic reaction. Moreover, mixed meals, uncertain culprit ingredients, concomitant medications, physical exercise, alcohol consumption, fasting, and intercurrent infection may modify the clinical response or complicate identification of the principal trigger [7–9]. The clinical presentation reflects the concurrence of a systemic hypersensitivity reaction and acute coronary involvement. Cutaneous and mucosal manifestations may include pruritus, erythema, urticaria, and angioedema, whereas respiratory, gastrointestinal, and hemodynamic involvement may manifest as dyspnea, wheezing, vomiting, abdominal pain, hypotension, or syncope. Cardiac manifestations include chest pain, palpitations, ischemic electrocardiographic abnormalities, myocardial injury, arrhythmias, and, in severe cases, cardiac arrest [1, 5, 7, 8]. Nevertheless, chest pain or cutaneous involvement may be absent despite objective evidence of myocardial ischemia or a severe systemic reaction. The absence of typical skin findings therefore does not exclude anaphylaxis or KS when cardiovascular manifestations develop in an appropriate temporal context [1, 5, 7, 8]. Diagnosis requires integration of the clinical chronology, electrocardiographic findings, cardiac biomarkers, mast-cell mediators, and cardiovascular imaging [1, 4, 5, 7, 8, 10]. Electrocardiographic abnormalities may include ST-segment elevation or depression, T-wave inversion, conduction disturbances, or ventricular arrhythmias, whereas serial cardiac troponin measurements can identify myocardial injury. Troponin elevation during an acute hypersensitivity reaction should prompt appropriate cardiovascular assessment but does not independently establish KS in the absence of other evidence supporting myocardial ischemia [1, 9, 10]. Acute serum tryptase, obtained within the appropriate temporal window and compared whenever possible with a subsequent baseline concentration, may support systemic mast-cell activation. However, a normal acute tryptase concentration does not exclude anaphylaxis, particularly in food-associated reactions [9]. Coronary angiography is particularly relevant for distinguishing transient vasospasm or nonobstructive coronary disease from fixed obstructive lesions, plaque disruption, intracoronary thrombosis, and stent thrombosis, thereby supporting classification of the KS phenotype. Coronary computed tomography angiography, intracoronary imaging, and cardiac magnetic resonance imaging may provide complementary mechanistic information in selected patients [1, 4, 5, 7, 8].

Once the patient has stabilized, a structured allergological assessment is important for evaluating the suspected food trigger and distinguishing the likely culprit from concomitant exposures or cofactors. The assessment should begin with a detailed chronology of food intake, symptom onset, physical exercise, alcohol consumption, fasting, intercurrent infection, and medication exposure. Skin prick testing with standardized extracts or prick-to-prick testing with the suspected food may demonstrate IgE sensitization when interpreted together with the clinical history. Measurement of food-specific IgE and, when clinically appropriate, component-resolved diagnostics may provide additional support for causal attribution, particularly in cofactor-dependent conditions such as wheat-dependent exercise-induced anaphylaxis [9, 11]. Basophil activation testing may offer complementary evidence in selected patients when conventional investigations do not resolve the diagnostic uncertainty. Nevertheless, sensitization alone does not establish clinical causality, and neither a positive skin test nor detectable food-specific IgE confirms that the sensitized food caused the coronary event [9, 11]. Oral food challenge may help resolve diagnostic uncertainty in food allergy but should not be performed routinely after a severe reaction involving myocardial ischemia. Any consideration of controlled oral exposure requires an individualized risk-benefit assessment, specific informed consent, and a closely monitored setting with appropriate cardiovascular and resuscitation support [12]. The temporal relationship between epinephrine administration and coronary manifestations also requires cautious interpretation. Intramuscular epinephrine remains the first-line treatment for anaphylaxis and should not be delayed when anaphylaxis is clinically suspected [5, 13, 14]. Coronary manifestations may precede its administration, become objectively documented only afterward, or worsen during the evolving hypersensitivity reaction. Temporal precedence alone cannot establish that epinephrine caused the coronary event because the underlying anaphylactic reaction may itself induce coronary vasospasm, myocardial ischemia, plaque destabilization, and thrombosis [5, 13, 14]. Epinephrine chronology should therefore be documented explicitly without being interpreted as proof of causality. Despite increasing recognition of KS, the evidence on food-associated presentations remains fragmented. Allergological investigation, cardiovascular characterization, measurement of mast-cell mediators, identification of cofactors, and reporting of treatment and follow-up are inconsistent across published cases. Previous reviews have predominantly examined KS across all triggering exposures, leaving the clinical and mechanistic spectrum of food-associated cases insufficiently characterized [1, 5, 7, 8]. Against this background, the present study reports an index case of food-associated KS and provides a systematic qualitative synthesis of the published evidence. The objectives were to characterize the reported food exposures and mechanistic categories, clinical and cardiovascular presentations, allergological and mast-cell assessments, potential cofactors, KS phenotypes and diagnostic certainty, management strategies, and reported outcomes. Sensitivity analyses were additionally undertaken to evaluate the consistency of the principal findings after restriction to the confirmed or suspected food-allergy stratum, restriction to patients classified as having definite or probable KS, and exclusion of patients in whom epinephrine preceded the onset or first objective documentation of coronary manifestations.

## Clinical Case Report

A 56-year-old man with established ischemic heart disease was evaluated after an acute episode suggestive of food-associated Kounis syndrome. His cardiovascular risk factors included hypertension, dyslipidemia, and former smoking, without diabetes mellitus. In March 2012, he underwent percutaneous revascularization of the proximal left anterior descending coronary artery. In September 2012, two drug-eluting stents were implanted because of proximal coronary stenosis. The manufacturer, model, dimensions, and drug platform of these previously implanted stents could not be retrieved from the available clinical records. His medical history also included lung cancer treated by right middle and lower lobectomy, without subsequent chemotherapy or radiotherapy.

Since 2018, he had experienced recurrent episodes of presumed histaminergic angioedema, averaging three to four episodes per year, together with symptomatic dermographism treated with bilastine 20 mg as needed. Additional allergic comorbidities included atopic dermatitis and allergic rhinoconjunctivitis associated with sensitization to cypress, olive, and grass pollens. He also reported an immediate hypersensitivity reaction to metamizole (Nolotil^®^), consisting of scalp pruritus and wheals developing within minutes of ingestion. He had previously tolerated non-pyrazolone nonsteroidal anti-inflammatory drugs (NSAIDs). His regular medication at the time of the index episode included acetylsalicylic acid 100 mg, esomeprazole 40 mg, ezetimibe 10 mg, and bilastine 20 mg as needed.

At midday on the day of the acute episode, he ate a hamburger prepared with white bread and undercooked beef and concomitantly took ibuprofen 400 mg. Approximately 2 h later, he developed generalized urticaria, facial angioedema, and tachycardia of up to 160 beats/min, with a blood pressure of 100/60 mmHg. He self-administered four 20-mg tablets of bilastine, corresponding to a total dose of 80 mg, after which he experienced somnolence. No other adverse effects attributable to bilastine were documented, and the somnolence did not require specific treatment. Approximately 3 h after the onset of the allergic manifestations, while the cutaneous symptoms were beginning to improve, he developed acute chest pain radiating to the right upper extremity. Upon arrival at the emergency department, the urticaria was already resolving and chest pain predominated. Given his history of coronary artery disease, the initial assessment focused on excluding pulmonary embolism and acute myocardial ischemia, and Kounis syndrome was not initially suspected. By the time of evaluation, the cutaneous manifestations were subsiding and the clinical presentation was dominated by chest pain, without ongoing respiratory compromise or documented shock. Epinephrine was therefore not administered. Electrocardiography showed sinus tachycardia without acute ischemic changes. Chest pain began at 20:30, and the first blood sample for cardiac troponin measurement was obtained at 21:17, corresponding to an interval of 47 min. Serial measurements demonstrated an initial high-sensitivity cardiac troponin T concentration of 35.23 ng/L, which decreased to 23.99 ng/L after 4 h and to 15.97 ng/L after 8 h (reference value < 14 ng/L), indicating dynamic myocardial injury. Serum tryptase reached 27.30 µg/L during the acute episode and subsequently decreased to 11.00 µg/L. A later baseline measurement was 5.90 µg/L, confirming a significant transient increase consistent with systemic mast-cell activation. Subsequent baseline measurements remained within the reference range. Renal function and serum electrolyte concentrations were preserved, while C-reactive protein and creatine kinase concentrations were within their respective reference ranges. The complete blood count showed mild leukocytosis of 12.5 × 10⁹/L, with neutrophilia of 7.93 × 10⁹/L. A markedly elevated D-dimer concentration of 8,960 ng/mL prompted evaluation for pulmonary embolism. Urgent computed tomography pulmonary angiography excluded pulmonary embolism. Transthoracic echocardiography showed no pericardial effusion or significant regional wall-motion abnormalities. A loading dose of ticagrelor was initially administered as part of the management of suspected acute coronary syndrome. During coronary angiography, after the coronary anatomy had been defined, the interventional cardiologist elected to administer a 60-mg loading dose of prasugrel. The specific rationale for switching P2Y12 inhibitors was not further documented in the available clinical record.

Coronary angiography demonstrated a dominant right coronary artery of normal caliber, patent previously implanted stents in the left anterior descending coronary artery without evidence of restenosis, and a critical stenosis containing a radiolucent thrombotic lesion in the proximal left circumflex coronary artery. Percutaneous coronary intervention was performed, with successful revascularization and implantation of a 3.5 × 13 mm Orsiro^®^ sirolimus-eluting stent (Biotronik, Berlin, Germany), achieving an optimal angiographic result. No intracoronary imaging with optical coherence tomography or intravascular ultrasound was performed during the intervention. No histological analysis of the thrombotic material was performed.

Three days after the acute event, molecular allergy testing using the ALEX2 macroarray (Allergy Xplorer, Macro Array Diagnostics) detected marked sensitization to ω−5 gliadin (Tri a 19), with a specific IgE concentration of 19.29 kUA/L. Sensitization was also detected to grass pollen Phl p 5 (5.25 kUA/L), cypress pollen Cup a 1 (2.05 kUA/L), cat allergen Fel d 1 (3.18 kUA/L), and olive pollen Ole e 1 (3.07 kUA/L).

At the 2-week follow-up, skin prick testing was performed with commercial extracts of gliadin, gluten, and wheat flour (Diater, Madrid, Spain), together with prick-to-prick testing using native wheat flour and gluten flour. Skin prick testing with the commercial gliadin extract was positive, with a wheal diameter of 6 × 4 mm, whereas the commercial wheat and gluten extracts yielded negative results. Prick-to-prick testing was positive with gluten flour (6 × 5 mm) and wheat flour (3 × 2 mm). The histamine positive control produced a wheal of 4 × 3 mm. These findings demonstrated sensitization to wheat-related allergens, in agreement with the clinical history and molecular results. ImmunoCAP testing (Thermo Fisher Scientific, Uppsala, Sweden), performed at the same visit, confirmed specific IgE concentrations of 1.08 kUA/L to wheat, 20.6 kUA/L to ω−5 gliadin, 9.62 kUA/L to gluten, and 6.46 kUA/L to gliadin. Specific IgE to α-Gal was undetectable (0.00 kUA/L), and total serum IgE was 161 kU/L. Serum tryptase returned to a baseline concentration of 5.90 µg/L and remained within the reference range during subsequent follow-up, with values ranging from 5.77 to 7.35 µg/L (reference value < 11.4 µg/L). Peripheral blood analysis by polymerase chain reaction and Sanger sequencing of exon 17 of the KIT gene did not detect the D816V mutation. TPSAB1 copy-number analysis for hereditary α-tryptasemia was not performed because basal serum tryptase concentrations consistently remained below 8 µg/L. The repeatedly normal and stable basal tryptase concentrations made a persistently elevated basal tryptase phenotype less likely, although hereditary α-tryptasemia could not be formally excluded without TPSAB1 copy-number analysis. Together with the negative peripheral blood KIT D816V result, these findings did not support an underlying clonal mast-cell disorder.

The temporal relationship between wheat ingestion, ibuprofen exposure, the systemic allergic reaction, and the subsequent acute coronary event, together with the cardiovascular and allergological findings, supported a diagnosis of type II Kounis syndrome associated with IgE-mediated wheat allergy and predominant sensitization to ω−5 gliadin. Type II KS was assigned because the event occurred in a patient with established coronary atherosclerotic disease and was characterized by a new thrombotic lesion in the left circumflex coronary artery. The patency of the previously implanted left anterior descending coronary stents argued against type III KS. At this stage, ibuprofen was considered either a competing trigger or a potential cofactor because tolerance to the drug in isolation had not yet been demonstrated. The patient was initially advised to follow a gluten-free diet and to avoid NSAIDs other than acetylsalicylic acid prescribed as antiplatelet therapy until completion of the allergological evaluation. Acetylsalicylic acid 100 mg was maintained throughout follow-up, including during all subsequent drug and food challenges. The patient was prescribed an epinephrine autoinjector and received education regarding its indications and use.

On September 4, 2023, a supervised oral drug challenge with ibuprofen was performed after obtaining specific written informed consent for drug provocation testing. The signed consent form was incorporated into the patient’s electronic medical record. The patient continued acetylsalicylic acid 100 mg as antiplatelet therapy throughout the procedure. Baseline electrocardiography, cardiac troponin, serum tryptase, and vital signs were obtained, and venous access was established. Continuous electrocardiographic monitoring was maintained throughout the procedure, and vital signs were reassessed with each dose increase. The patient remained under clinical observation after completion of the challenge. A cumulative dose of 600 mg of ibuprofen was tolerated without allergic or cardiovascular manifestations, and the challenge was considered negative. This result demonstrated tolerance to ibuprofen when administered in isolation and permitted the reintroduction of non-pyrazolone NSAIDs. In the context of the index reaction, ibuprofen was therefore considered a probable cofactor of the wheat-associated allergic reaction rather than the primary allergenic trigger. However, the negative challenge did not reproduce the original combined exposure and therefore could not definitively establish the contribution of ibuprofen to the index event.

Subsequent evaluation confirmed negative celiac serology. Skin testing to gliadin and wheat remained positive, although wheat-related specific IgE concentrations showed a decreasing trend. On April 2, 2024, a supervised oral food challenge with 250 g of cooked pasta, corresponding to approximately 10 g of gluten, was performed after obtaining specific written informed consent for food provocation testing. The signed consent form was incorporated into the patient’s electronic medical record. The patient continued acetylsalicylic acid 100 mg as antiplatelet therapy throughout the procedure. Baseline electrocardiography, cardiac troponin, serum tryptase, and vital signs were obtained, and venous access was established. Continuous electrocardiographic monitoring was maintained throughout the procedure, and vital signs were reassessed with each dose increase. The patient remained under clinical observation after completion of the challenge. The full dose was tolerated in the absence of additional relevant cofactors, without allergic or cardiovascular manifestations. The negative pasta challenge demonstrated clinical tolerance of the administered dose under the specific conditions of the procedure but did not exclude wheat allergy or reactions occurring at a different exposure threshold or in the presence of relevant cofactors. On the basis of this result, complete avoidance of traces or cross-contamination was not considered necessary. The patient was advised that larger wheat exposures and combinations with potential cofactors, particularly NSAIDs other than his established low-dose acetylsalicylic acid treatment and physical exercise, could not be considered safe solely on the basis of the negative challenge.

After the pasta challenge, the patient initially consumed six cookies daily but discontinued regular gluten intake after approximately 1 week because of flatulence, abdominal distension, loose stools, and pharyngeal irritation. These manifestations were not accompanied by immediate cutaneous, respiratory, hemodynamic, or cardiovascular symptoms and were not considered consistent with an immediate IgE-mediated reaction. He consequently resumed a predominantly gluten-free diet without strict avoidance of traces or cross-contamination.

On September 18, 2024, a second supervised oral food challenge was performed after obtaining specific written informed consent for food provocation testing. The signed consent form was incorporated into the patient’s electronic medical record. The patient continued acetylsalicylic acid 100 mg as antiplatelet therapy throughout the procedure. Baseline electrocardiography, cardiac troponin, serum tryptase, and vital signs were obtained, and venous access was established. Continuous electrocardiographic monitoring was maintained throughout the procedure, and vital signs were reassessed with each dose increase. The patient remained under clinical observation after completion of the challenge. Two María cookies, corresponding to approximately 700 mg of gluten, were tolerated without allergic or cardiovascular manifestations. Given the demonstrated tolerance of a full serving of pasta and a smaller amount of gluten-containing cookies under supervised conditions and in the absence of additional relevant cofactors, complete gluten avoidance was not considered necessary. Regular consumption of a small tolerated amount of gluten was initially recommended to maintain exposure at the demonstrated tolerated level and avoid unnecessarily strict dietary restriction. This recommendation did not imply that unrestricted wheat consumption or exposure in the presence of relevant cofactors was considered safe. HLA genotyping for celiac disease susceptibility was negative for both HLA-DQ2 and HLA-DQ8.

At the most recent documented follow-up in June 2025, the patient remained free of recurrent urticaria, angioedema, anaphylaxis, and new coronary events. He reported following a predominantly gluten-free diet without strict avoidance of traces or cross-contamination. He continued to tolerate small quantities of gluten, such as a piece of bread or a cookie, frequently but not necessarily every day, and occasionally consumed a portion of gluten-containing pasta. These exposures were tolerated without immediate allergic or cardiovascular manifestations. Nevertheless, the absence of recurrence during these exposures was not considered to exclude cofactor-dependent wheat allergy or to establish the safety of unrestricted wheat consumption. He continued to carry an epinephrine autoinjector and remained under joint allergological and cardiological follow-up.

## Systematic Qualitative Synthesis of Published Case Reports and Case Series

### Information Sources and Search Strategy

A systematic literature search was conducted in PubMed, Scopus, and Web of Science Core Collection from database inception to August 22, 2026, without a lower publication-date restriction. The search combined terms related to Kounis syndrome and allergic coronary presentations with generic and food-specific exposure terms, including anaphylaxis, allergic angina, allergic myocardial infarction, food ingestion, food allergy, fish, shellfish, wheat, nuts, fruit, mushrooms, Anisakis, histamine poisoning, and scombroid syndrome. The complete database-specific search strategies, search interfaces, search dates, and numbers of records retrieved are provided in the Supplementary Methods. Additional reports were sought through backward reference checking of the included publications and relevant reviews, forward citation searching, and targeted web searches for recent, non-indexed, or conference publications. Reports identified through these supplementary methods were assessed using the same eligibility criteria as database-derived records. Eighteen additional eligible publications were identified through supplementary searching, including eight through backward reference checking, six through forward citation searching, and four through targeted web searching.

### Record Management and Study Selection

**Fig. 1:**
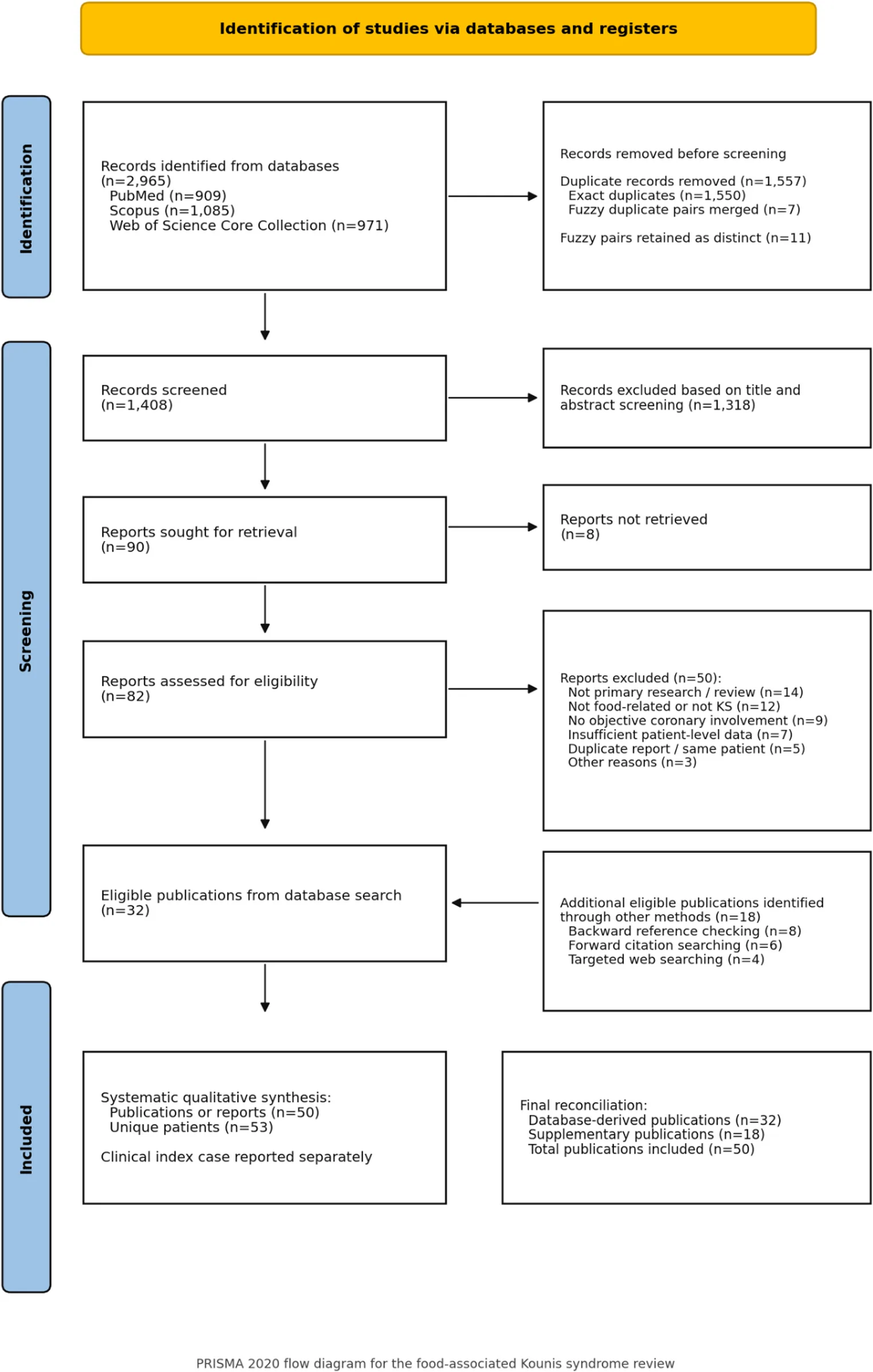
PRISMA 2020 study-identification and selection process

The database searches retrieved 2,965 records: 909 from PubMed, 1,085 from Scopus, and 971 from Web of Science Core Collection. All records retrieved from the three databases were imported into Covidence (Veritas Health Innovation, Melbourne, Australia) for record management, deduplication, and study selection. Covidence-assisted deduplication was supplemented by manual verification using normalized DOI, PMID, database accession information, and titles. Records with identical or highly similar titles were reviewed manually to distinguish true duplicates from article-reply pairs, online-first and final versions, conference abstracts, and separate publications with similar titles. Overall, 1,557 duplicate records were removed, comprising 1,550 exact duplicates and seven records merged after manual adjudication of potential fuzzy duplicates. Eleven potential fuzzy duplicate pairs were retained as distinct records after manual review. This process left 1,408 unique database records for title and abstract screening. Two reviewers independently screened titles and abstracts against the predefined eligibility criteria. A total of 1,318 records were excluded at this stage, and 90 reports were sought for full-text retrieval. Eight reports could not be retrieved, leaving 82 reports for full-text eligibility assessment. Fifty reports were excluded after full-text assessment: 14 were not primary research or were review articles, 12 were not food-related or did not meet the criteria for Kounis syndrome, nine lacked objective coronary involvement, seven provided insufficient patient-level information, five were duplicate reports or described an already included patient, and three were excluded for other reasons. Thirty-two eligible publications were retained from the database search. An additional 18 eligible publications were identified through backward reference checking, forward citation searching, and targeted web searching, and were assessed using the same eligibility criteria. The final synthesis therefore included 50 publications or reports describing 53 unique patients. Disagreements at either stage were resolved through discussion and, when consensus could not be reached, by consultation with a third reviewer. Study selection and reporting followed the PRISMA 2020 statement [15], and the fully reconciled selection process is presented in Fig. 1.

The publication and the patient were treated as separate units of analysis. Multiple publications describing the same patient were linked and counted as a single patient, whereas case series contributed the number of independently identifiable patients with food-associated KS. Duplicate physical copies or bibliographic records of the same publication were counted only once. When two or more publications potentially described the same patient, overlap was assessed using the author group, publication year, country and clinical center, patient age and sex, food exposure, clinical chronology, coronary phenotype, angiographic findings, treatment, and outcome. Potential overlaps were evaluated by two reviewers, and unresolved cases were discussed with a third reviewer. When reports were judged to describe the same patient, they were linked and treated as a single patient-level observation, while complementary information from each report was retained.

### Eligibility Criteria

Eligible publications described at least one original patient in whom objective myocardial ischemia, acute coronary syndrome, coronary vasospasm, plaque-related myocardial infarction, or stent or graft thrombosis occurred in temporal association with ingestion of a food or food-derived exposure. Eligible cardiovascular evidence comprised ischemic electrocardiographic abnormalities, cardiac biomarker elevation accompanied by other findings supporting myocardial ischemia, new regional wall-motion abnormalities, coronary vasospasm, angiographic plaque disruption or thrombosis, or stent or graft thrombosis. Troponin elevation during a hypersensitivity reaction was not considered sufficient by itself to establish KS when objective evidence of myocardial ischemia was absent or the clinical course and cardiovascular investigations supported a more plausible alternative explanation. The clinical index case presented in this article was analyzed separately and was not included in the systematic synthesis.

Reviews, editorials, and commentaries without original patient data; mechanistic studies; pharmacovigilance analyses; reports involving exclusively non-food triggers; publications with insufficient patient-level information; and duplicate or secondary descriptions of an already included patient were excluded. Conference abstracts were eligible only when they contained sufficient information to establish the food exposure, hypersensitivity presentation, cardiovascular event, and independence of the reported patient. No publication was excluded solely because of language, journal-indexing status, or the absence of formal allergological testing. Publication type, source characteristics, and reporting completeness were considered in the methodological appraisal and interpretation of the findings.

### Data Extraction

Patient-level data were extracted by one reviewer using a standardized and piloted data-extraction form and were independently verified against the original publications by a second reviewer. Discrepancies were resolved through discussion and re-examination of the source report, with consultation of a third reviewer when consensus could not be reached. Multiple reports potentially describing the same patient were examined jointly before incorporation into the dataset. After completion of verification, the consolidated patient-level dataset was cross-checked against Tables 1A and 2B, and all aggregate counts and denominators presented in Tables 3, 4 and 5 were recalculated from the verified patient-level dataset. Extracted variables included publication characteristics, age, sex, cardiovascular history and risk factors, suspected food exposure, latency from ingestion to symptom onset, potential cofactors or contributing exposures, allergic manifestations, cardiovascular symptoms, electrocardiographic findings, cardiac biomarkers, echocardiography, coronary angiography, coronary computed tomography angiography, intracoronary imaging, cardiac magnetic resonance imaging, serum tryptase, food-specific IgE, component-resolved diagnostics, skin testing, provocation or re-exposure procedures, treatment, outcome, author-reported KS phenotype, reviewer-derived phenotype, KS diagnostic certainty, and certainty of food causality. Treatment variables included antihistamines, systemic corticosteroids, epinephrine, the timing and route of epinephrine administration, coronary vasodilators, antiplatelet agents, anticoagulation, percutaneous coronary intervention, thrombus aspiration, cardiopulmonary resuscitation, and other cardiovascular or hemodynamic support. Treatment was coded only when the specific intervention was explicitly identifiable in the publication. Unreported treatment was retained as not reported and was not interpreted as absence.

**Table 1:** A. Demographic, Exposure, Clinical, and Cardiological Characteristics of Published Patients With Food-Associated Kounis Syndrome

Study	Design	Patient	Food-associated exposure	Mechanistic category	Cofactor, contributing exposure, or relevant condition	Time to onset	Clinical manifestations	Cardiac findings	KS phenotype	KS diagnostic certainty	Reference
Dogan et al., 2019	Case report	M, 67 y	Blue crab	Clinically suspected food allergy	None reported	1 h after ingestion	Precordial chest pain, pruritus, facial urticarial rash, palpitations, mild respiratory distress, tachypnea, and expiratory wheezing	Inferior ST-segment elevation in leads II, III, and aVF; inferior wall hypokinesia; LVEF 55%; elevated troponin I and CK-MB; coronary angiography without coronary atherosclerosis	Type I, author reported	Probable	[61]
Gargiulo et al., 2021	Case report	M, 53 y	Tuna, probable scombroid poisoning	Scombroid poisoning (non immune)	None reported	Shortly after ingestion; exact latency NR	Tongue swelling, syncope, hypotension	Inferior ST elevation; transmural ischemic lesion on CMR; nonocclusive mid RCA thrombus on OCT	Type II, author reported	Definite	[28]
Clemen et al., 2021	Case report	M, 67 y	Nut-containing bar	Clinically suspected food allergy	Known nut allergy; established coronary artery disease and previous coronary stent implantation; epinephrine administered before chest pressure and its first objective cardiac documentation	Shortly after ingestion; exact latency NR	Urticaria, tongue and oropharyngeal angioedema, dyspnea, tachypnea, diaphoresis, nausea, and subsequent intermittent chest pressure	Intermittent chest pressure; ECG without ST changes; mid inferolateral hypokinesia; patent previous stent with approximately 30% in-stent restenosis and a 95% ostial lesion in a jailed diagonal branch	Type II, author reported	Probable	[42]
De Gennaro et al., 2017	Case report	M, 57 y	Canned tuna	Scombroid poisoning (non immune)	None reported	After canned tuna ingestion; exact latency NR	Facial rash, angioedema, palpitations, hypotension, collapse	Initial diffuse ST depression followed by inferior ST elevation; normal coronary angiography; presumed transient spasm	Type I, reviewer derived	Probable	[24]
Dogan et al., 2018	Case report	M, 17 y	BCAA supplement	Clinically suspected food allergy	None reported	30 min after ingestion	Chest pain, generalized pruritus, facial rash, palpitations	Inferolateral ST elevation; inferior wall hypokinesia; normal coronary angiography	Type I, author reported	Probable	[62]
Tepetam et al., 2016	Case report	F, 31 y	Pleurotus ostreatus mushroom	Confirmed food allergy	None reported	2 h	Nausea, abdominal pain, vomiting, dyspnea, chest pain	Anterolateral ST elevation; LV dysfunction; diffuse proximal LAD narrowing resolving after intracoronary nitrate	Type I, author reported	Definite	[44]
Toya et al., 2019	Case report	F, 62 y	Mango	Confirmed food allergy	Physical exercise; aspirin- associated exacerbation; epinephrine preceded reported ischemic worsening	Several hours after ingestion, during exercise	Urticaria, angioedema, dyspnea, wheezing, abdominal pain, and chest tightness	ST depression and T-wave inversion; cardiac biomarkers not elevated; no obstructive coronary artery disease; acetylcholine induced distal RCA spasm	Type I, author reported	Definite	[31]
Tzanis et al., 2017	Case report	M, 70 y	Rice pudding containing sheep milk	Confirmed food allergy	Recently implanted LAD stent; evening medication taken approximately 20 min earlier	Allergic symptoms during ingestion; chest pain approximately 15 min later	Generalized urticaria, pruritus, lip angioedema, and chest pain	Anterior ST elevation; acute thrombosis of recently implanted LAD stent	Type III, author- reported	Definite	[37]
Wada et al., 2010	Case report	F, 65 y	Noodles containing shellfish	Clinically suspected food allergy	None reported	30 min	Pruritus, rash, dizziness, sweating, vomiting, hypotensive shock; no chest pain	Transient inferior ST elevation and AV block; cardiac biomarkers not elevated; normal baseline coronary angiography; ergometrine-induced complete mid-RCA spasm	Type I, author- reported	Definite	[46]
Yaegashi et al., 2011	Case report	M, 70 y	Wheat noodles	Clinically suspected food allergy	Physical exercise after wheat ingestion; chronic aspirin treatment	Urticaria during exercise approximately 2 h after ingestion; chest pain approximately 1 h after exercise	Urticaria, chest pain	Anterolateral ST elevation; Thrombotic proximal LAD occlusion with residual organic stenosis after thrombus aspiration.	Type II, reviewer- derived	Definite	[32]
González-de- Olano et al., 2012, patient 1	Case series	M, 42 y	Gilthead fish	Clinically suspected food allergy	Underlying nonclonal mast- cell activation syndrome	NR	Urticaria, hypotension, syncope, and oppressive chest pain	ST elevation; normal coronary angiography	Type I, reviewer- derived	Probable	[20]
González-de- Olano et al., 2012, patient 2	Case series	M, 62 y	Lentil	Clinically suspected food allergy	Underlying clonal mast-cell activation syndrome	NR	Hypotension, syncope and oppressive chest pain	ECG without acute ischemic abnormalities; nonobstructive coronary atheromatosis	Type II, reviewer- derived	Probable	[20]
Cuevas-Bravo et al., 2022	Case series	M, 87 y	Shellfish	Confirmed food allergy	Underlying ischemic cardiomyopathy and hypertension	Minutes	Generalized urticaria, abdominal pain, diarrhea, hypotension, tachycardia, wheezing, hypoxemia, chest pain	Diffuse ST depression with ST elevation in aVR; angiography NP due to comorbidity	Type II, author- reported	Probable	[48]
Cesarz and Ganti 2023	Case report	M, 41 y	Grapes	Clinically suspected food allergy	Epinephrine administered during anaphylaxis	Minutes	Facial Angioedema, hypotension, gastrointestinal manifestations, chest pain	Transient ST-segment elevation in V1-V3; normal left ventricular systolic function without regional wall-motion abnormalities;	Type I, author-reported	Possible	[63]
Rodríguez-Ruiz et al., 2019	Case report	F, 23 y	Milk	Clinically suspected food allergy	Known milk allergy; accidental exposure	Sudden onset	Dyspnea, palpitations, chest discomfort	Mild LV dysfunction; coronary anatomy insufficiently reported for reliable phenotype assignment	Unclassifiable	Possible	[64]
Wajtryt et al., 2019	Case report	M, 35 y	Nut mixture	Confirmed food allergy	None reported	Rapid; exact latency NR	Urticaria, syncope, hypotension, and transient atrial fibrillation	Transient inferior ST-segment elevation with subsequent T-wave inversion in leads III and aVF	Type I, reviewer- derived	Probable	[65]
Reinhold et al., 2023	Case report	F, 56 y	Banana	Confirmed food allergy	Recurrent exposure	Recurrent symptoms; exact latency NR	Oral pruritus and angina	Positive stress ECG; clinically suspected coronary vasospasm	Type I, reviewer- derived	Possible	[66]
Lin et al., 2021	Case report	F, 58 y	Raw fish gallbladder	Clinically suspected food allergy	Biphasic anaphylaxis	30 min	Angioedema, urticaria, flushing, gastrointestinal manifestations, hypotension	nitial lateral ST-segment depression and T-wave abnormalities followed by inferior ST-segment elevation; near-complete RCA occlusion due to vasospasm, reversed by intracoronary nitroglycerin	Type I, author reported	Definite	[47]
Xu and Wang, 2022	Case report	M, 70 y	Ice cream	Clinically suspected food allergy	None reported	Approximately 20 min	Syncope and chest pain	Delayed ST elevation myocardial infarction; LAD spasm relieved by diltiazem; intracoronary imaging without relevant atherosclerosis	Type I, reviewer derived	Definite	[45]
Al Hariri et al., 2025	Case report	M, 41 y	Fish meal	Clinically suspected food allergy	None reported	20 min	Generalized pruritus, erythematous skin lesions, chest pain, palpitations, and dizziness	Initial diffuse ST-segment depression with ST-segment elevation in aVR, followed by inferior ST-segment elevation; myocardial injury; obstructive proximal LCx disease	Type II, author reported	Probable	[67]
Xu and Han et al., 2024	Case report	M, 34 y	Oysters	Clinically suspected food allergy	None reported	2 h	Urticaria, severe chest pain	ST-segment elevation myocardial infarction, ventricular fibrillation, and LAD occlusion	Type II, author reported	Definite	[35]
Watanabe et al., 2020	Case report	M, 47 y	Wheat bread	Confirmed food allergy	Epinephrine before the onset or first objective documentation of coronary manifestations	Allergic symptoms 18 min after ingestion	Urticaria, shock; chest pain after epinephrine	ST-segment elevation and acute thrombosis of a previously implanted LAD stent	Type III, author reported	Definite	[38]
Vaina et al., 2019	Case report	F, 62 y	Green salad	Clinically suspected food allergy	Culprit ingredient not established	Approximately 20 min	Urticaria, vomiting, syncope, dyspnea, hypotension	Inferolateral ST-segment elevation with reciprocal changes; normal coronary angiography	Type I, author reported	Probable	[21]
Jariwala et al., 2022	Case report	M, 67 y	Mushroom-containing food	Clinically suspected food allergy	Previous CABG and previously stented saphenous vein graft	Reported as approximately 24 h	Generalized urticaria and chest pain	ST depression; very late stent thrombotic in-stent occlusion of a saphenous vein graft, multivessel coronary disease, and refractory no-reflow	Type III, author reported; proposed type IV also discussed	Definite	[36]
Gázquez et al., 2010	Case series	M, 66 y	Kiwi	Confirmed food allergy	None reported	NR	Urticaria, vomiting, hypotension, chest pain	ST depression; angiography NR	Unclassifiable	Possible	[68]
Rosier et al., 2018	Case report	M, 49 y	Wheat	Confirmed food allergy	Physical exercise after wheat ingestion	During exercise after wheat ingestion; exact interval NR	Urticaria, angioedema, abdominal pain, vomiting, diarrhea, hypotension, and collapse	Prehospital ST-segment elevation; no regional wall-motion abnormality; no structural coronary disease identified on subsequent cardiac assessment	Type I, reviewer derived	Probable	[33]
Yang et al., 2023	Case series	M, 40 y	Seafood	Clinically suspected food allergy	Epinephrine before chest pain	Chest pain approximately 20 min after epinephrine	Allergic manifestations after seafood ingestion followed by chest pain	ST elevation in aVR with diffuse ST depression; rapid ECG normalization; coronary findings reported as nonobstructive/normal in the source summary	Type I, author reported	Possible	[50]
Armstrong et al., 2025	Case report	M, 23 y	Peanut	Confirmed food allergy	Epinephrine before worsening ECG abnormalities	Approximately 30 min after ingestion	Throat symptoms, rash, vomiting, and chest pain	Bradycardia, ventricular bigeminy, marked ischemic ST-segment abnormalities, and normal echocardiography; coronary angiography not reported	Type I, reviewer derived	Probable	[51]
Hoxha and Ndreu et al., 2025	Case report	M, 43 y	Tomato salad	Confirmed food allergy	Probable nsLTP mediated food Allergy; Previously implanted LAD stent	Approximately 30 min	Generalized urticaria, facial angioedema, throat discomfort, and syncope	Acute thrombosis of a previously implanted LAD stent	Type III, author reported	Definite	[41]
Hamideh et al., 2025	Case series	F, 48 y	Suspected shellfish exposure	Clinically suspected food allergy	Food causality and epinephrine contribution uncertain	Chest tightness developed within minutes after epinephrine	Pre-epinephrine allergic manifestations were not clearly documented; chest tightness radiating to the left shoulder and jaw	ST elevation and myocardial injury; normal echocardiography and coronary angiography	Type I, author reported	Possible	[22]
Gomez-Olarte et al., 2025, patient 2	Case series	M, 73 y	Tilapia, papaya, and borojó; individual culprit food not established	Clinically suspected food allergy	Mixed food exposure and simultaneous amoxicillin-clavulanate exposure	NR	Edema, pruritus, dyspnea, dizziness, and chest pain	NSTEMI progressing to inferior STEMI; ulcerated proximal RCA lesion with a large thrombus burden; mast cells identified in aspirated thrombus	Type II, reviewer derived	Definite	[23]
Tan et al., 2026	Case report	M, 53 y	Dragon fruit	Clinically suspected food allergy	Cardiovascular risk factors and underlying coronary atherosclerosis	Approximately 1 h	Generalized pruritus, dyspnea, nausea, vomiting, diffuse abdominal pain, wheezing, and hypoxemia; no chest pain	Dynamic ischemic T-wave abnormalities; inferolateral hypokinesis; critical mid- RCA stenosis requiring PCI	Type II, author reported	Probable	[56]
Zavras et al., 2003, patient 1	Case series	F, 55 y	Shellfish	Clinically suspected food allergy	None reported	Allergic symptoms approximately 1 h after ingestion; myocardial infarction approximately 2 h later	Generalized rash, pruritus, dyspnea, nausea, and vomiting, followed by severe chest pain, diaphoresis, hypotension, and bradycardia	ST elevation; 80% proximal LAD lesion with angioplasty and stenting	Type II, author reported	Definite	[52]
Zavras et al., 2003, patient 2	Case series	M, 50 y	Shellfish	Clinically suspected food allergy	Epinephrine administered before chest pain and first objective coronary manifestations	Immediate urticaria; chest pain while in the emergency department	Urticaria, vomiting, faintness, hypotension, and bradycardia, followed by central chest pain	Inferior ST-segment elevation; moderate RCA stenosis treated with angioplasty	Type II, author reported	Definite	[52]
Coppola et al., 2012	Case report	M, 56 y	Tuna-associated scombroid poisoning	Scombroid poisoning (non immune)	None reported	Immediate	Facial rash, dysgeusia, headache, diaphoresis, and severe hypotension	Dynamic ST-segment depression followed by inferior/right-sided ST-segment elevation; coronary vasospasm together with a critical LAD lesion	Type II, reviewer derived	Probable	[25]
Barbarroja Escudero et al., 2013	Case report	M, 59 y	Fish eggs contaminated with Anisakis simplex	Confirmed food allergy	Cardiovascular risk factors: smoking, diabetes mellitus, and dyslipidemia	Chest pain approximately 30 min after the allergic manifestations	Burning mouth, urticaria, lip angioedema, vomiting, dyspnea, and chest pain	Anterior ST-segment elevation; marked myocardial injury; multivessel coronary atherosclerosis with an 80% LAD lesion treated with a drug-eluting stent	Type II, author reported	Definite	[69]
Guler et al., 2014	Case report	M, 23 y	Kiwifruit	Clinically suspected food allergy	Previous oral allergy syndrome after kiwifruit	After ingestion; exact latency not reported	Diffuse erythema, rash, pruritus, and chest pain	Inferior ST elevation; preserved ventricular function, and normal coronary angiography	Type I, author reported	Definite	[70]
Nachmias and Leibowitz et al., 2014	Case report	F, 59 y	Salad with mustard dressing	Clinically suspected food allergy	Cardiovascular risk factors: hypertension, smoking, and hyperlipidemia	Immediate	Generalized warmth, palpitations, diffuse erythema, urticaria, and light-headedness; no chest pain	Transient anterior ST-segment elevation with reciprocal depression; normal ventricular function and coronary angiography	Type I, reviewer derived	Possible	[71]
Mejía-Rentería et al., 2014	Case report	M, 44 y	Undercooked fish contaminated with Anisakis simplex	Confirmed food allergy	Pre-existing eosinophilia; epinephrine administered after the onset of chest pain and coronary manifestations	Within minutes after ingestion	Anaphylactic shock, hives, vomiting, hypotension	Transient inferior ST elevation; normal angiography/OCT; normal left ventricular systolic function	Type I, author reported	Definite	[53]
Valle Alonso et al., 2015	Case report	M, 27 y	*Coprinopsisatramentaria* plus beer	Non-immune toxic reaction (Coprinopsis alcohol interaction)	Alcohol consumption; asthma, nasal polyposis, and aspirin hypersensitivity	After mushroom and alcohol ingestion; exact interval NR; chest pain 5 min after hospital arrival	Dizziness, vomiting, chest pain, diaphoresis, urticaria, syncope, hypotension, and bradycardia	Inferior ST-segment elevation with reciprocal changes and third-degree atrioventricular block; mid-inferior left ventricular hypokinesia; normal coronary angiography	Type I, author reported	Probable	[30]
Cucunato et al., 2015	Case report	F, 28 y	Tuna-associated scombroid poisoning	Scombroid poisoning (non immune)	Psoriasis, insulin resistance, arterial hypertension, and venous insufficiency	Approximately 1 h	Nausea, vomiting, diaphoresis, marked weakness, and persistent hypotension	Lateral repolarization abnormalities consistent with subendocardial ischemia; coronary angiography showed no coronary artery disease	Type I, reviewer derived	Probable	[26]
Michas et al., 2017	Case report	M, 49 y	Mushrooms	Clinically suspected food allergy	Drug-eluting LAD stent implanted 2 weeks earlier; recent anterior STEMI; former smoking	Dyspnea and urticaria after mushroom ingestion; acute coronary syndrome 12 h later	Dyspnea and urticaria, followed by squeezing retrosternal chest pain radiating to the left arm	Acute anterior ST-segment elevation myocardial infarction due to thrombosis of a recently implanted stent; restoration of TIMI 3 flow after thrombectomy and balloon dilatation	Type III, author-reported	Definite	[57]
Rusconi and Villa, 2017	Case report	F, 59 y	Tuna; probable scombroid poisoning	Scombroid poisoning (non-immune)	NR	Immediately after tuna ingestion; exact latency NR	Flushing, diffuse erythematous rash, asthenia, palpitations, and burning sensation in the mouth; no chest pain	Sinus tachycardia with diffuse ST-segment depression, followed by rapid normalization; presumed transient coronary vasospasm	Type I, reviewer-derived	Possible	[27]
Fiore et al., 2021	Case report	M, 52 y	Almonds	Clinically suspected food allergy	Epinephrine administered after the onset of cardiovascular manifestations and the first objective documentation of myocardial ischemia	Approximately 10 min after ingestion	Transient loss of consciousness, lower thoracic or epigastric pain, nausea, vomiting, diaphoresis, and intense tremors	Sinus tachycardia; mild ST-segment elevation in V1–V3, hyperacute precordial T waves, and reciprocal ST-segment depression in the inferior and lateral leads; normal biventricular function without regional wall-motion abnormalities; CCTA showed no significant obstructive epicardial coronary disease, with only mild LAD atherosclerosis	Type I, author-reported	Probable	[54]
Acosta and Elfessi, 2023	Case report	M, 57 y	Rice containing shrimp	Confirmed food allergy	Known shellfish allergy; hypertension, hyperlipidemia, recurrent pulmonary embolism, and stage 3 chronic kidney disease	Approximately 30 min	Tongue and hard-palate swelling, hoarseness, dyspnea, exertional chest pain, diaphoresis, and shortness of breath	ST-segment elevation myocardial infarction; coronary imaging without a fixed obstructive lesion or thrombosis	Type I, reviewer-derived	Probable	[55]
Foti Randazzese et al., 2024	Case report	M, 14 y	Medlar	Confirmed food allergy	Previous oral symptoms after cherry and apple ingestion	A few minutes after ingestion	Facial angioedema, urticaria, and chest pain	Nonspecific ventricular repolarization abnormalities; normal echocardiography; coronary anatomy not assessed	Type I, reviewer-derived	Probable	[49]
Ali et al., 2025	Case report	M, 53 y	Peanut butter and grapefruit juice; peanut subsequently identified as the culprit	Confirmed food allergy	Grapefruit juice as a concomitant exposure, subsequently excluded by allergy testing	Shortly after ingestion; exact interval NR	Generalized pruritus, urticaria, facial and lip angioedema, central chest tightness, dyspnea, and presyncope;	Progressive ST-segment depression and deep T-wave inversion with troponin elevation; coronary angiography without significant obstructive disease; cardiac magnetic resonance imaging without myocardial infarction	Type I, author-reported	Probable	[72]
Chardin et al., 2025, patient 1	Case series	M, 50 y	Wheat-containing food	Confirmed food allergy	Physical exercise; amoxicillin taken 2 days earlier and subsequently excluded as the trigger; epinephrine administered after the onset of chest pain and myocardial ischemia	Allergic and cardiovascular manifestations developed after 15 min of endurance exercise, approximately 2 h after wheat ingestion	Urticaria, abdominal pain, nausea, dyspnea, uvular edema, hypotension, and chest pain	Inferior ST-segment elevation; right coronary artery occlusion on coronary angiography	Type I, author-reported;	Definite	[34]
Chardin et al., 2025, patient 2	Case series	F, 58 y	Wheat-pasta	Confirmed food allergy	Hypertension, dyslipidemia, and previously implanted coronary stent	Urticaria approximately 1 h after wheat-pasta ingestion during hospitalization	Initial chest pain followed by urticaria	Initial ST-segment elevation; coronary angiography demonstrated intrastent occlusion	Type III, author reported	Probable	[34]
Pelagatti et al., 2025	Case report	F, 59 y	Improperly stored canned tuna	Scombroid poisoning (non immune)	Improper food storage; arterial hypertension; epinephrine administered before the first objective documentation of coronary manifestations	Generalized rash and pruritus approximately 30 min after ingestion; chest pain documented on emergency department arrival approximately 5 h after ingestion	Pruritus, erythema, diarrhea, chest pain radiating to the interscapular region, severe hypotension, tachycardia, tachypnea, and acute respiratory distress	Diffuse ST-segment depression in V3-V6, I, II, III, aVL, and aVF, with ST-segment elevation in aVR; high-sensitivity troponin T elevation; normal biventricular function and preserved wall motion; coronary CT angiography without significant coronary stenosis	Type I, author−reported	Probable	[29]
Monerris Aguilar et al., 2026	Case report	M, 61 y	Mustard/Sin a 2	Confirmed food allergy	Underlying coronary artery disease; two intramuscular epinephrine doses administered before chest pain	Immediately after ingestion; chest pain developed approximately 1 h later	Palmoplantar pruritus, generalized urticaria, lingual angioedema, hypotension, and chest pain	Normal ECG; regional hypokinesia on echocardiography; NSTEMI, Killip class II; coronary anatomy and mechanism not reported	Type II, reviewer derived	Probable	[43]
Fisher et al., 2026	Case report	M, 58 y	Seafood	Clinically suspected food allergy	Metabolic syndrome and coronary atherosclerosis; intramuscular epinephrine and infusion administered before the first ischemic ECG	Shortly after ingestion; exact interval NR	Angioedema, hypotension, respiratory distress	ST-segment elevation in the anterolateral and inferior leads; angiography demonstrated 95% mid-LAD stenosis caused by plaque rupture with superimposed thrombus, with mild atherosclerotic disease in the circumflex and right coronary arteries; normal left ventricular systolic function	Type II, author reported	Definite	[40]
Arends et al., 2022	Conference abstract	M, 61 y	Accidental peanut ingestion	Confirmed food allergy	Known peanut allergy; smoking, diabetes, coronary disease. Epinephrine before reported cardiovascular worsening	Allergic manifestations after accidental ingestion; exact latency NR	Urticaria, pruritus, hypotension, chest pain, myocardial ischemia, and pulseless ventricular tachycardia	Initial inferolateral ST-segment elevation; coronary angiography followed by LAD and RCA stent implantation; recurrent ST-segment elevation after PCI, progressing to pulseless ventricular tachycardia and requiring revascularization and intra-aortic balloon pump support	Type II with subsequent suspected type III, author-reported	Probable	[39]

*NR* not reported, *NP* not performed. KS phenotypes were classified as author-reported or reviewer-derived. The definite, probable, and possible categories represent a review-specific adjudication framework and are not externally validated diagnostic criteria for Kounis syndrome. Abbreviations: *AV* atrioventricular, *BCAA* branched-chain amino acids, *CAD* coronary artery disease, *CCTA* coronary computed tomography angiography, *CMR* cardiac magnetic resonance, *DES* drug-eluting stent, *ECG* electrocardiogram, *KS* Kounis syndrome, *LAD* left anterior descending coronary artery, *LCx* left circumflex coronary artery, *LVEF* left ventricular ejection fraction, *NP* not performed, *NR* not reported, *nsLTP* nonspecific lipid transfer protein, *OCT* optical coherence tomography, *PCI* percutaneous coronary intervention, *RCA* right coronary artery, *STEMI* ST-segment elevation myocardial infarction, *VT* ventricular tachycardia

**Table 2:** B. Laboratory, Allergological, Treatment, and Outcome Characteristics of Published Patients With Food- Associated Kounis Syndrome

Study	Food-allergy evidence or non-immune mechanism	Serum tryptase measured	Paired acute/baseline tryptase	Troponin status	Coronary imaging performed	Fixed obstructive coronary lesion or thrombosis	Epinephrine chronology	Antiallergic treatment	Cardiovascular treatment and support	Outcome	Reference
Dogan et al., 2019	Clinical/uncertain	Yes; 54.5 µg/L	No	Elevated	Yes	No	NR	Antihistamine and cortico steroids	NR	Resolution of ECG and echocardiographic abnormalities by day 6	[61]
Gargiulo et al., 2021	Non-immune scombroid poisoning	No	No	Elevated	Yes	Yes	NR	Antihistamine	Aspirin; subsequent antithrombotic management	Favorable recovery	[28]
Clemen et al., 2021	Clinical/uncertain	No	No	Elevated	Yes	Yes	Administered before the onset or first objective documentation of coronary manifestations	Three doses of epinephrine, antihistamine and cortico steroids	Aspirin, heparin infusion and atorvastatin,	Chest pain resolved and troponin concentrations decreased; discharged with cardiology follow-up	[42]
De Gennaro et al., 2017	Non-immune scombroid poisoning	No	No	Elevated	Yes	No	NR	Antihistamine and cortico steroids	Aspirin, nitrates, atorvastatin, and pantoprazole	Recovery, Uneventful 6- month follow- up	[24]
Dogan, 2018	Clinical/uncertain	Yes; 51 µg/L	No	Elevated	Yes	No	NR	Antihistamine and cortico steroids	NR	Complete ECG, biomarker and echocardiographic resolution by day 4	[62]
Tepetam et al., 2016	Confirmed	Yes	No	Elevated	Yes	No	NR	NR	Coronary vasodilator treatment	Asymptomatic at 3 months	[44]
Toya et al., 2019	Confirmed	No	No	Not elevated	Yes	No	Administered after allergic onset and before reported worsening of ischemic manifestations	Epinephrine; other antiallergic treatment NR	NR	No recurrence at 1 year	[31]
Tzanis et al., 2017	Confirmed	Yes	No	NR	Yes	Yes	NR	NR	PCI/coronary intervention	PCI and favorable recovery	[37]
Wada et al., 2010	Clinical/uncertain	No	No	Not elevated	Yes	No	NR	NR	NR	No recurrence after discharge	[46]
Yaegashi et al., 2011	Clinical/uncertain	No	No	Elevated (biomarker-confirmed myocardial infarction)	Yes	Yes	NR	NR	Thrombus aspiration	Discharged without residual stenosis	[32]
González de-Olano et al., 2012, patient 1	Clinical/uncertain	Yes	Yes	Not elevated	Yes	No	NR	NR	NR	Favorable outcome	[20]
González de-Olano et al., 2012, patient 2	Clinical/uncertain	Yes	No	Not elevated	Yes	No	NR	NR	NR	Favorable outcome	[20]
Cuevas Bravo et al., 2022	Confirmed	Yes	Yes	Elevated	No	NR	NR	NR	NR	Favorable outcome	[48]
Cesarz and Ganti, 2023	Clinical/uncertain	No	No	Not elevated	No	NR	Administered before the first objective documentation of coronary manifestations	Epinephrine	NR	Favorable outcome	[63]
Rodríguez Ruiz et al., 2019	Clinical/uncertain	No	No	Elevated	No	NR	NR	NR	NR	Favorable outcome	[64]
Wajtryt et al., 2019	Confirmed	Yes	No	Elevated	No	NR	NR	NR	NR	Favorable outcome	[65]
Reinhold et al., 2023	Confirmed	Yes	No	Not elevated	Yes	No	NR	NR	NR	Clinical resolution after banana avoidance	[66]
Lin et al., 2021	Clinical/uncertain	No	No	Not elevated	Yes	No	Administered after coronary manifestations and before the ischemic worsening	Antihistamine, famotidine and cortico steroids	Coronary vasodilator treatment	Favorable outcome	[47]
Xu andWang, 2022	Clinical/uncertain	No	No	Elevated	Yes	No	NR	NR	Coronary vasodilator treatment	Favorable outcome	[45]
Al Hariri et al., 2025	Clinical/uncertain	No	No	Elevated	Yes	Yes	NR	NR	Coronary angiography and treatment of obstructive coronary disease;	Troponin decreased from 461 to 168 ng/L; favorable recovery	[67]
Xu and Han, 2024	Clinical/uncertain	No	No	Elevated	Yes	Yes	NR	NR	PCI/coronary intervention; cardiopulmonary resuscitation	Successful resuscitation and coronary intervention	[35]
Watanabe et al., 2020	Confirmed	No	No	Not elevated initially	Yes	Yes	Administered before the onset or first objective documentation of coronary manifestations	Epinephrine; other antiallergic treatment incompletely reported	PCI, thrombus aspiration, repeat coronary intervention, and mechanical circulatory support	Successful treatment of acute stent thrombosis; discharged with wheat avoidance recommendations	[38]
Vaina et al., 2019	Clinical/uncertain	No	No	Elevated	Yes	No	NR	Antihistamine and corticosteroid	Airway and hemodynamic support, including intubation and norepinephrine	Favorable outcome; full recovery	[21]
Jariwala et al., 2022	Clinical/uncertain	No	No	Elevated	Yes	Yes	NR	NR	Thrombus aspiration, coronary vasodilator treatment, PCI	PCI complicated by refractory no-reflow, followed by stabilization and discharge	[36]
Gázquez et al., 2010	Confirmed	Yes	No	Elevated	No	NR	NR	NR	NR	Complete recovery	[68]
Rosier et al., 2018	Confirmed	Yes	No	Elevated	NR	No	NR	Epinephrine and antihistamine	NR	Subsequent wheat-exercise re-exposure reproduced allergic symptoms; favorable outcome with avoidance of relevant cofactors	[33]
Yang et al., 2023	Clinical/uncertain	No	No	Not elevated	Yes	No	Administered before the onset or first objective documentation of coronary manifestations	Epinephrine; antihistamine and corticosteroid	Nitrate or coronary vasodilator treatment reported	Rapid ECG normalization and favorable outcome	[50]
Armstrong et al., 2025	Confirmed	No	No	Elevated	No	NR	Administered before the onset or first objective documentation of coronary manifestations	Epinephrine, antihistamine and corticosteroid	NR	Favorable recovery	[51]
Hoxha and Ndreu, 2025	Confirmed	No	No	NR	Yes	Yes	NR	NR	PCI/coronary intervention	Successful PCI and favorable recovery	[41]
Hamideh et al., 2025	Clinical/uncertain	No	No	Elevated	Yes	No	Administered before the onset or first objective documentation of coronary manifestations	Epinephrine; other antiallergic treatment not clearly identifiable	Sublingual nitroglycerin	Symptoms resolved; stable discharge after observation	[22]
Gomez Olarte et al., 2025, patient 2	Clinical/uncertain	Yes	No	NR	Yes	Yes	NR	NR	Thrombus aspiration and PCI with stent implantation	Successful revascularization and favorable recovery	[23]
Tan et al., 2026	Clinical/uncertain	No	No	Elevated	Yes	Yes	Epinephrine was administered during initial anaphylaxis treatment	Epinephrine, antihistamine and corticosteroid	PCI with an everolimus-eluting stent to the mid-RCA	Resolution of the wall-motion abnormality at 3 months; no allergic or cardiac events at 1 year	[56]
Zavras et al., 2003, patient 1	Clinical/uncertain	Yes	No	Elevated	Yes	Yes	NR	Antihistamine and corticosteroid	Thrombolysis, angioplasty, and coronary stenting	Uneventful recovery	[52]
Zavras et al., 2003, patient 2	Clinical/uncertain	Yes	No	Elevated	Yes	Yes	Administered before the onset or first objective documentation of coronary manifestations	Epinephrine, antihistamine and corticosteroid	Angioplasty/coronary intervention	Uneventful recovery	[52]
Coppola et al., 2012	Non-immune scombroid poisoning	No	No	Elevated	Yes	Yes	NR	Antiallergic/supportive treatment reported but incompletely characterized	Coronary vasodilator treatment and PCI of the LAD lesion	Recovered	[25]
Barbarroja Escudero et al., 2013	Confirmed	Yes, baseline only	No	Elevated	Yes	Yes	No epinephrine administered before coronary manifestations	Antihistamine and corticosteroid	Fibrinolysis followed by PCI with drug-eluting stent implantation	No subsequent allergic or cardiac recurrence reported	[69]
Guler et al., 2014	Clinical/uncertain	No	No	Elevated	Yes	No	NR	Parenteral corticosteroid, fluids, and antihistamine	NR	Recovered	[70]
Nachmias and Leibowitz, 2014	Clinical/uncertain	No	No	Not elevated	Yes	No	Not administered	Systemic corticosteroid	Aspirin, clopidogrel, and low-molecular-weight heparin	Recovered; subsequent skin-prick testing did not identify a specific allergen	[71]
Mejía Rentería et al., 2014	Confirmed	No	No	Elevated	Yes	No	Administered after the onset of chest pain and the first objective documentation of coronary manifestations	Epinephrine, systemic corticosteroid, antihistamine	Calcium-channel blocker prescribed at discharge and subsequently discontinued after confirmation of the allergic trigger	Complete clinical and ECG resolution; discharged after 72 h; asymptomatic at 6-month follow-up after avoiding undercooked fish	[53]
Valle Alonso et al., 2015	Other non-immune toxic reaction	Yes	Yes	Elevated	Yes	No	Administered before the onset or first objective documentation of coronary manifestations	Epinephrine, systemic corticosteroid antihistamine	Oxygen, clopidogrel, and low-molecular-weight heparin	Recovered	[30]
Cucunato et al., 2015	Non-immune scombroid poisoning	No	No	Elevated	Yes	No	NR	NR	NR	Recovered	[26]
Michas et al., 2017	Clinical/uncertain	No	No	NR	Yes	Yes	Not administered	Systemic corticosteroid and antihistamine	Aspiration thrombectomy and PCI with balloon; aspirin and clopidogrel	Discharged 10 days later; recovered	[57]
Rusconi and Villa, 2017	Non-immune scombroid poisoning	No	No	NR	No	NR	Not administered	Antihistaminecorticosteroid and famotidine	NR	Rapid resolution	[27]
Fiore et al., 2021	Clinical/uncertain	No	No	Elevated	Yes	No	Administered after the onset of cardiovascular manifestations and the first objective documentation of myocardial ischemia	Epinephrine, systemic corticosteroid antihistamine	Diltiazem prescribed at discharge	Discharged asymptomatic approximately 60 h after presentation; Recovered	[54]
Acosta and Elfessi, 2023	Confirmed	No	No	Elevated	Yes	No	Administered after the first objective documentation of coronary manifestations	Epinephrine, systemic corticosteroid antihistamine and Famotidine	Aspirin and emergent coronary angiography; no PCI performed	Recovered	[55]
Foti Randazzese et al., 2024	Confirmed	No	No	Elevated	No	NR	Not administered	Antihistamine and corticosteroid	NR	Complete clinical, electrocardiographic, and laboratory resolution within a few days; Resolved	[49]
Ali et al., 2025	Confirmed	Yes	No	Elevated	Yes	No	Not administered	Antihistamine and corticosteroid	Nitroglycerin, dual antiplatelet therapy, and low-molecular-weight heparin	Remained asymptomatic after peanut avoidance Recovered	[72]
Chardin et al., 2025, patient 1	Confirmed	Yes	Yes	Elevated	Yes	Yes	Administered after the onset of chest pain and the first objective documentation of myocardial ischemia	Epinephrine, corticosteroid antihistamine and fluid	Coronary angiography; no stenting required	Acute recovery; subsequent exercise-induced anaphylaxis due to nonadherence during follow-up	[34]
Chardin et al., 2025, patient 2	Confirmed	Yes, baseline only	No	Elevated	Yes	Yes	NR	NR	Coronary angiography documented intrastent occlusion	Symptom-free at 1-year follow-up while following a gluten-free diet	[34]
Pelagatti et al., 2025	Non-immune scombroid poisoning	No	No	Elevated	Yes	No	Administered before the first objective documentation of coronary manifestations	Continuous epinephrine infusion, corticosteroid antihistamine and fluid	noninvasive mechanical ventilation, and supplemental oxygen	Discharged home after approximately 3 days Recovered	[29]
Monerris Aguilar et al., 2026	Confirmed	Yes, baseline only	No	Elevated	No	NR	Two 0.5-mg intramuscular epinephrine doses administered before chest pain and the first objective cardiac documentation	Epinephrine, corticosteroid antihistamine and nebulized β2-agonists,	NR	Recovered Mustard avoidance, emergency anaphylaxis kit, and cardiology follow-up; subsequent clinical outcome NR	[43]
Fisher et al., 2026	Clinical/uncertain	No	No	Elevated/acute STEMI reported; numerical troponin value NR	Yes	Yes	Intramuscular epinephrine and subsequent epinephrine infusion administered before the first objective documentation of coronary manifestations	Three 0.2-mg intramuscular epinephrine doses, followed by epinephrine infusion; corticosteroidand antihistamine	Emergency coronary angiography and PCI with drug-eluting stent implantation in the mid-LAD; dual antiplatelet therapy and secondary cardiovascular prevention	Recovered Resolution of ST-segment elevation and hemodynamic stabilization after revascularization; preserved LVEF of 65%; discharged on hospital day 3	[40]
Arends, 2022	Confirmed	No	No	NR	Yes	Yes	Administered before the first objective documentation of coronary manifestations	Epinephrine, systemic corticosteroid antihistamine and famotidine	Initial LAD and RCA stent implantation, clopidogrel, cardiopulmonary resuscitation, repeat revascularization, intra-aortic balloon pump placement, discharged on prasugrel	Survived pulseless ventricular tachycardia and was discharged on prasugrel; longer-term follow-up NR	[39]

*NR* not reported, *NP* not performed. Mechanistic categories were mutually exclusive, and IgE mediation was considered demonstrated only when supported by allergological findings. KS phenotypes were classified as author-reported or reviewer-derived

**Table 3:** Summary of Demographic, Mechanistic, Cardiological, Allergological, and Phenotypic Findings

Variable	Patients, *n*/*N* (%) or summary	Additional information
Included evidence		
Publications or reports	50	Index case excluded
Unique patients	53	Patients described more than once were counted once
Demographic characteristics		
Age reported	53/53 (100.0%)	Median, 55 years; range, 14–87 years.
Male sex	39/53 (73.6%)	Reporting frequency among published patients.
Female sex	14/53 (26.4%)	Reporting frequency among published patients.
Food-related mechanism		
Confirmed food allergy	20/53 (37.7%)	Food allergy was considered confirmed when supported by the allergological assessment reported in Table 2B. IgE mediation was demonstrated only when supported by specific allergological findings.
Clinically suspected food allergy	26/53 (49.1%)	Includes clinically compatible food-associated reactions without sufficient evidence to confirm the culprit food or an IgE-mediated mechanism.
All confirmed or clinically suspected food allergy	46/53 (86.8%)	Combined food-allergy stratum used in the sensitivity analysis.
Scombroid poisoning	6/53 (11.3%)	Non-immune food poisoning analyzed separately from food allergy.
Other non-immune toxic reaction	1/53 (1.9%)	*Coprinopsis atramentaria*-alcohol interaction.
Cardiological findings		
ECG status reported	52/53 (98.1%)	ECG status was considered known when an acute ECG result or an explicit description of its absence was available.
ECG abnormality among patients with known status	49/52 (94.2%)	Included ischemic ST-segment or T-wave abnormalities, conduction disturbances, ventricular ectopy, and ventricular arrhythmias.
Troponin status reported or classifiable	47/53 (88.7%)	Included numerical results and explicit diagnosis-derived evidence of biomarker-confirmed myocardial infarction.
Troponin elevation among patients with known status	37/47 (78.7%)	Qualitative or diagnosis-derived elevation was distinguished from a directly reported numerical result.
Coronary imaging performed	43/53 (81.1%)	Included invasive coronary angiography, coronary CT angiography, and intracoronary imaging. Echocardiography, cardiac magnetic resonance imaging, and stress testing alone were not counted.
Fixed obstructive coronary lesion or thrombosis among patients with classifiable status	20/44 (45.5%)	Isolated or presumed vasospasm, normal coronary arteries, and nonobstructive coronary disease were not counted.
Allergological assessment		
Skin prick or prick-to-prick testing reported	11/53 (20.8%)	Included conventional skin-prick and prick-to-prick testing when explicitly reported.
Food-specific IgE or component-resolved diagnostics reported	19/53 (35.8%)	Total IgE alone was not counted.
Oral food challenge or documented oral food re-exposure	2/53 (3.8%)	Included one formal oral food challenge and one documented oral food re-exposure. Coronary provocation and exercise testing without oral food exposure were not counted.
Serum tryptase measured	20/53 (37.7%)	Included acute, baseline-only, and paired measurements when explicitly reported.
Paired acute and baseline tryptase measurements	4/53 (7.5%)	Only clearly identifiable acute and subsequent baseline measurements were counted.
Epinephrine chronology		
Epinephrine explicitly administered	20/53 (37.7%)	Includes Toya et al., in whom epinephrine preceded the reported ischemic worsening but did not clearly precede the initial coronary manifestations or their first objective documentation. Michas et al. was not included because epinephrine was not administered.
Epinephrine before the onset or first objective documentation of coronary manifestations	12/53 (22.6%)	Toya et al., Mejía-Rentería et al., Fiore et al., and Chardin et al. patient 1 were not included. Temporal precedence was not interpreted as evidence of causality.
KS diagnostic certainty		
Definite KS	21/53 (39.6%)	
Probable KS	24/53 (45.3%)	
Possible KS	8/53 (15.1%)	
KS phenotype		
Type I	29/53 (54.7%)	Includes author-reported and reviewer-derived type I classifications.
Type II	16/53 (30.2%)	Includes Arends, who was classified as type II with subsequent suspected type III KS.
Type III	6/53 (11.3%)	Arends was counted once within the type II category and was not counted again as type III.
Unclassifiable	2/53 (3.8%)	Insufficient cardiovascular information for reliable phenotype assignment.

Values represent reporting frequencies among published patients; the index case was excluded. Denominators vary according to data availability, and unknown or insufficiently reported findings were treated as missing rather than absent. Mechanistic categories were mutually exclusive. The 46-patient food-allergy stratum comprised 20 patients with confirmed food allergy and 26 with clinically suspected food allergy. IgE mediation was considered demonstrated only when supported by allergological findings. Toya et al. and Lin et al. were not classified as epinephrine-first cases because epinephrine preceded a reported ischemic worsening but did not precede the initial objective coronary manifestations. Mejía-Rentería et al., Fiore et al., and Chardin et al. patient 1 were not classified as epinephrine-first because coronary manifestations or their first objective documentation preceded epinephrine administration. Temporal precedence was descriptive and was not interpreted as evidence of causality

**Table 4:** Sensitivity Analyses of Principal Demographic, Cardiological, and Phenotypic Findings

Variable	All included patients (*n* = 53)	Confirmed or suspected food-allergy stratum (*n* = 46)	Definite or probable KS (*n* = 45)	Excluding epinephrine-first cases (*n* = 41)
Male sex	39/53 (73.6%)	35/46 (76.1%)	36/45 (80.0%)	30/41 (73.2%)
Acute ECG abnormalities	49/52 (94.2%)	42/45 (93.3%)	42/45 (93.3%)	39/40 (97.5%)
Troponin elevation	37/47 (78.7%)	31/41 (75.6%)	34/40 (85.0%)	30/36 (83.3%)
Fixed obstructive coronary lesion or thrombosis	20/44 (45.5%)	18/38 (47.4%)	20/40 (50.0%)	15/35 (42.9%)
Type I KS	29/53 (54.7%)	24/46 (52.2%)	23/45 (51.1%)	23/41 (56.1%)
Type II KS	16/53 (30.2%)	14/46 (30.4%)	16/45 (35.6%)	11/41 (26.8%)
Type III KS	6/53 (11.3%)	6/46 (13.0%)	6/45 (13.3%)	5/41 (12.2%)
Unclassifiable phenotype	2/53 (3.8%)	2/46 (4.3%)	0/45 (0.0%)	2/41 (4.9%)

Data are n/N (%). For ECG abnormalities, troponin elevation, and fixed obstructive coronary lesions or thrombosis, denominators include only patients with known or classifiable status within each analytical stratum. Sensitivity analyses excluded non-immune food-related reactions, possible KS cases, or epinephrine-first cases, as applicable. Toya et al. and Lin et al. were not considered epinephrine-first cases because epinephrine preceded a reported ischemic worsening but did not precede the initial objective coronary manifestations. Mejía-Rentería et al., Fiore et al., and Chardin et al. patient 1 were retained because coronary manifestations or their first objective documentation preceded epinephrine administration. Fisher et al. was classified as an epinephrine-first case because intramuscular epinephrine and the subsequent infusion preceded the first objective documentation of coronary manifestations. Analyses were descriptive, and no inferential comparisons were performed

**Table 5:** Summary of Reported Treatment and Outcomes Among Patients With Food-Associated Kounis Syndrome

Variable	Patients, *n*/*N* (%)	Additional information
Antiallergic treatment		
Epinephrine explicitly administered	20/53 (37.7%)	Includes Toya et al., in whom epinephrine preceded the reported ischemic worsening but did not clearly precede the initial coronary manifestations or their first objective documentation. Michas et al. was not counted because epinephrine was not administered.
Epinephrine before the onset or first objective documentation of coronary manifestations	12/53 (22.6%)	Toya et al., Mejía-Rentería et al., Fiore et al., and Chardin et al. patient 1 were not included. Temporal precedence was descriptive and was not interpreted as evidence that epinephrine caused the coronary event.
Cardiovascular treatment and support		
Any explicitly identifiable cardiovascular treatment, intervention, or support	32/53 (60.4%)	Included explicitly reported coronary medication, antithrombotic treatment, therapeutic coronary intervention, vasopressor or hemodynamic support, respiratory support, and cardiopulmonary resuscitation. Diagnostic coronary angiography alone was not counted unless accompanied by treatment or support.
PCI or other therapeutic coronary intervention	15/53 (28.3%)	Included angioplasty, stent implantation, balloon dilatation, repeat revascularization, and other explicitly reported therapeutic coronary procedures.
Thrombus aspiration	5/53 (9.4%)	Reported in Yaegashi et al., Watanabe et al., Jariwala et al., Gomez-Olarte et al. patient 2, and Michas et al. Categories were not mutually exclusive.
Coronary vasodilator treatment	11/53 (20.8%)	Included explicitly reported nitrates, diltiazem, other calcium-channel blockers, or intracoronary vasodilators administered acutely or prescribed for the presumed vasospastic mechanism.
Cardiopulmonary resuscitation	2/53 (3.8%)	Reported in Xu and Han and Arends.
Outcomes		
No fatal outcome reported	53/53 (100.0%)	No fatal outcomes were reported in the included publications. This should not be interpreted as an estimate of survival or prognosis because of publication bias, selective outcome reporting, and incomplete follow-up.
Follow-up of at least 3 months explicitly reported	6/53 (11.3%)	Documented in De Gennaro et al., Tepetam et al., Toya et al., Tan et al., Mejía-Rentería et al., and Chardin et al. patient 2.

Data are n/N (%) among the 53 published patients. Treatments were counted only when explicitly identifiable, and categories were not mutually exclusive. NR was not interpreted as absence. The category “any explicitly identifiable cardiovascular treatment, intervention, or support” included pharmacological cardiovascular treatment, therapeutic coronary procedures, hemodynamic or respiratory support, and cardiopulmonary resuscitation, but not diagnostic testing or monitoring alone. Epinephrine chronology was descriptive and did not establish causality. Toya et al. and Lin et al. were not classified as epinephrine-first cases because epinephrine preceded a reported ischemic worsening but did not precede the initial objective coronary manifestations. Mejía-Rentería et al., Fiore et al., and Chardin et al. patient 1 were not included because coronary manifestations or their first objective documentation preceded epinephrine administration. Temporal precedence was descriptive and was not interpreted as evidence of causality

Qualitative descriptions of latency, such as “shortly after ingestion,” “rapidly,” or “soon after,” were retained as qualitative information and were not assigned automatically to quantitative intervals. Troponin was classified as elevated when the reported value exceeded the reference limit provided in the publication or when the authors explicitly documented biomarker-confirmed myocardial injury or myocardial infarction. Acute and baseline tryptase measurements were considered paired only when the publication clearly identified a sample obtained during or shortly after the acute reaction and a subsequent baseline measurement. Skin prick or prick-to-prick testing, food-specific IgE, component-resolved diagnostics, and oral food challenge or documented oral food re-exposure were recorded separately. Total IgE alone was not considered evidence of food-specific sensitization. Pharmacological coronary provocation and exercise testing without oral food exposure were not classified as food-challenge evidence. Epinephrine was classified as preceding the coronary event only when it was administered before the onset of cardiovascular manifestations or before the first objective documentation of myocardial ischemia. Cases in which coronary manifestations had already begun before epinephrine administration were not included in this category. When epinephrine preceded a subsequent worsening of ischemic manifestations but did not clearly precede their initial onset or first objective documentation, this chronology was recorded separately. Temporal precedence was not interpreted as evidence that epinephrine caused the coronary event.

### Diagnostic and Causal Adjudication

For the purposes of this review, the authors developed a review-specific adjudication framework to classify KS diagnostic certainty as definite, probable, or possible. These categories were used to structure the qualitative assessment of published cases and should not be interpreted as externally validated diagnostic criteria for Kounis syndrome. KS diagnostic certainty, KS phenotype, food causality, and the presumed biological mechanism were assessed as separate dimensions. KS diagnostic certainty was categorized as definite, probable, or possible according to the temporal relationship between the hypersensitivity event and the cardiovascular manifestations; the presence of objective ischemic evidence on electrocardiography, cardiac biomarkers, echocardiography, cardiac magnetic resonance imaging, coronary angiography, or intracoronary imaging; the demonstration of coronary vasospasm, plaque disruption, thrombosis, or coronary findings compatible with a transient spastic event; and the presence of plausible alternative explanations. A definite diagnosis required a compatible acute hypersensitivity reaction and objective evidence of an acute coronary event, together with coronary or intracoronary findings directly supporting vasospasm, plaque disruption, coronary thrombosis, or stent or graft thrombosis, without a more plausible alternative diagnosis. A probable diagnosis required a compatible temporal relationship between a hypersensitivity reaction and objective myocardial ischemia, although the proposed coronary mechanism was not demonstrated directly. A possible diagnosis was assigned when objective cardiovascular evidence was limited, an important competing diagnosis remained, or the available chronology did not permit reliable distinction between a hypersensitivity-associated coronary event and another plausible mechanism. Epinephrine administration alone did not determine diagnostic certainty.

The KS phenotype was classified as type I, type II, type III, or unclassifiable. Type I comprised coronary vasospasm or compatible transient myocardial ischemia in patients without fixed obstructive coronary disease. Type II comprised vasospasm, plaque disruption, myocardial infarction, or coronary thrombosis in patients with underlying atherosclerotic coronary disease. Type III comprised hypersensitivity-associated thrombosis or occlusion of a previously implanted coronary stent. Graft thrombosis involving a previously stented coronary bypass graft was retained as type III, while the proposed type IV phenotype was recorded when discussed by the original authors. Cases with insufficient cardiovascular information were classified as unclassifiable. The phenotype stated by the original authors and the phenotype derived during the present review were recorded separately, and reviewer-derived classifications were considered presumptive when coronary imaging was absent or incomplete. Normal coronary angiography was not interpreted as excluding type I KS, particularly when angiography was performed after antiallergic treatment, nitrates, or other coronary vasodilators, because transient vasospasm may have resolved before coronary assessment. Food-related mechanisms were categorized as confirmed or suspected food allergy, scombroid poisoning caused by exogenous histamine, or another non-immune food-related toxic reaction. These categories were mutually exclusive. An IgE-mediated mechanism was considered demonstrated only when supported by compatible allergological findings and was not inferred solely from the clinical presentation. Evidence of sensitization alone was not considered sufficient to establish clinical food allergy. Food causality was assessed according to the clinical chronology, compatibility of the reaction, previous tolerance or reactions, allergological findings, oral challenge or re-exposure information, presence of cofactors, and assessment of alternative triggers. Mixed meals, unresolved culprit ingredients, and simultaneous medication exposures were retained explicitly rather than attributed to a specific food without adequate supporting evidence.

### Methodological Appraisal and Reporting Completeness

Because the evidence consisted predominantly of case reports and small case series, methodological appraisal was performed using the design-specific Joanna Briggs Institute critical appraisal checklists [16, 17]. Case reports were assessed using the eight-item JBI Critical Appraisal Checklist for Case Reports, whereas case series were assessed using the ten-item JBI Critical Appraisal Checklist for Case Series. Each item was rated as yes, no, unclear, or not applicable. Because the appraisal criteria differed according to study design, item-level judgments were interpreted using the corresponding checklist and were not combined into a numerical summary score. No quality threshold was used to exclude publications from the qualitative synthesis.

Reporting completeness was considered descriptively with reference to relevant CARE reporting domains, including clinical chronology, diagnostic assessment, treatment rationale, adverse events, follow-up, and patient perspective. This assessment was not interpreted as methodological quality, risk of bias, or certainty of evidence. Because no prospectively defined scoring rule or validated threshold was applied, publications were not classified into summary reporting categories. Information that could not be identified explicitly was classified conservatively as unclear rather than absent, and the conference abstract was interpreted separately because several reporting domains could not be assessed from an abstract-only source. All eligible publications were retained in the qualitative synthesis. Publication-level JBI judgments are presented in Table 6.

**Table 6:** JBI Methodological Appraisal and CARE Reporting Assessment of Included Case Reports and Case Series

Study	Design	Q1	Q2	Q3	Q4	Q5	Q6	Q7	Q8	Q9	Q10	Overall appraisal	Reference
Dogan et al., 2019	Case report	Y	Y	Y	Y	U	Y	U	Y	NA	NA	Retained	[61]
Gargiulo et al., 2021	Case report	Y	Y	Y	Y	U	Y	U	Y	NA	NA	Retained	[28]
Clemen et al., 2021	Case report	Y	Y	Y	Y	Y	Y	U	Y	NA	NA	Retained	[42]
De Gennaro et al., 2017	Case report	Y	U	Y	Y	U	Y	U	Y	NA	NA	Retained	[24]
Dogan, 2018	Case report	Y	Y	Y	Y	U	Y	U	Y	NA	NA	Retained	[62]
Tepetam et al., 2016	Case report	Y	Y	Y	Y	Y	Y	U	Y	NA	NA	Retained	[44]
Toya et al., 2019	Case report	Y	Y	Y	Y	Y	Y	U	Y	NA	NA	Retained	[31]
Tzanis et al., 2017	Case report	Y	Y	Y	Y	Y	Y	Y	Y	NA	NA	Retained	[37]
Wada et al., 2010	Case report	Y	Y	Y	Y	U	Y	Y	Y	NA	NA	Retained	[46]
Yaegashi et al., 2011	Case report	Y	Y	Y	Y	Y	Y	U	Y	NA	NA	Retained	[32]
González de-Olano et al., 2012	Case series	U	Y	Y	U	U	Y	Y	Y	U	Y	Retained	[20]
Cuevas Bravo et al., 2022	Case series	U	Y	Y	U	U	Y	Y	Y	U	Y	Retained	[48]
Cesarz and Ganti, 2023	Case report	Y	Y	Y	Y	U	Y	U	Y	NA	NA	Retained	[63]
Rodríguez Ruiz et al., 2019	Case report	Y	Y	Y	Y	U	Y	U	Y	NA	NA	Retained	[64]
Wajtryt et al., 2019	Case report	U	Y	Y	Y	U	Y	U	Y	NA	NA	Retained	[65]
Reinhold et al., 2023	Case report	Y	Y	Y	Y	U	Y	U	Y	NA	NA	Retained	[66]
Lin et al., 2021	Case report	Y	Y	Y	Y	U	Y	U	Y	NA	NA	Retained	[47]
Xu and Wang, 2022	Case report	Y	Y	Y	Y	U	Y	U	Y	NA	NA	Retained	[45]
Al Hariri et al., 2025	Case report	Y	Y	Y	Y	U	Y	U	Y	NA	NA	Retained	[67]
Xu and Han, 2024	Case report	Y	Y	Y	Y	Y	Y	Y	Y	NA	NA	Retained	[35]
Watanabe et al., 2020	Case report	Y	Y	Y	Y	Y	Y	Y	Y	NA	NA	Retained	[38]
Vaina et al., 2019	Case report	Y	Y	Y	Y	U	Y	U	Y	NA	NA	Retained	[21]
Jariwala et al., 2022	Case report	Y	Y	Y	Y	Y	Y	Y	Y	NA	NA	Retained	[36]
Gázquez et al., 2010	Case series	U	Y	Y	U	U	Y	Y	Y	U	Y	Retained	[68]
Rosier et al., 2018	Case report	Y	Y	Y	Y	U	Y	U	Y	NA	NA	Retained	[33]
Yang et al., 2023	Case series	U	Y	Y	U	U	Y	Y	Y	U	Y	Retained	[50]
Armstrong et al., 2025	Case report	Y	Y	Y	Y	Y	Y	U	Y	NA	NA	Retained	[51]
Hoxha and Ndreu, 2025	Case report	Y	Y	Y	Y	Y	Y	Y	Y	NA	NA	Retained	[41]
Hamideh et al., 2025	Case series	U	Y	Y	U	U	Y	Y	Y	U	Y	Retained	[22]
Gomez Olarte et al., 2025	Case series	U	Y	Y	U	U	Y	Y	Y	U	Y	Retained	[23]
Tan et al., 2026	Case report	Y	Y	Y	Y	Y	Y	U	Y	NA	NA	Retained	[56]
Zavras et al., 2003	Case series	U	Y	Y	U	U	Y	Y	Y	U	Y	Retained	[52]
Coppola et al., 2012	Case report	Y	Y	Y	Y	Y	Y	U	Y	NA	NA	Retained	[25]
Barbarroja Escudero et al., 2013	Case report	Y	Y	Y	Y	U	Y	U	Y	NA	NA	Retained	[69]
Guler et al., 2014	Case report	Y	Y	Y	Y	U	Y	U	Y	NA	NA	Retained	[70]
Nachmias and Leibowitz, 2014	Case report	Y	Y	Y	Y	U	Y	U	Y	NA	NA	Retained	[71]
Mejía Rentería et al., 2014	Case report	Y	Y	Y	Y	Y	Y	Y	Y	NA	NA	Retained	[53]
Valle Alonso et al., 2015	Case report	Y	Y	Y	Y	Y	Y	U	Y	NA	NA	Retained	[30]
Cucunato et al., 2015	Case report	Y	Y	Y	Y	U	Y	U	Y	NA	NA	Retained	[26]
Michas et al., 2017	Case report	Y	Y	Y	Y	Y	Y	Y	Y	NA	NA	Retained	[57]
Rusconi and Villa, 2017	Case report	Y	Y	Y	Y	U	Y	U	Y	NA	NA	Retained	[27]
Fiore et al., 2021	Case report	Y	Y	Y	Y	Y	Y	U	Y	NA	NA	Retained	[54]
Acosta and Elfessi, 2023	Case report	Y	Y	Y	Y	Y	Y	U	Y	NA	NA	Retained	[55]
Foti Randazzese et al., 2024	Case report	Y	Y	Y	Y	U	Y	U	Y	NA	NA	Retained	[49]
Ali et al., 2025	Case report	Y	Y	Y	Y	U	Y	U	Y	NA	NA	Retained	[72]
Chardin et al., 2025	Case series	U	Y	Y	U	U	Y	Y	Y	U	Y	Retained	[34]
Pelagatti et al., 2025	Case report	Y	Y	Y	Y	Y	Y	Y	Y	NA	NA	Retained	[29]
Monerris Aguilar et al., 2026	Case report	Y	Y	Y	Y	Y	Y	U	Y	NA	NA	Retained	[43]
Fisher et al., 2026	Case report	Y	Y	Y	Y	Y	Y	U	Y	NA	NA	Retained	[40]
Arends, 2022	Conference abstract	Y	Y	Y	Y	Y	Y	Y	U	NA	NA	Retained	[39]

*JBI* indicates Joanna Briggs Institute; *Y* yes, *N* no, *U* unclear, *NA* not applicable. JBI ratings were assigned conservatively. Case reports were assessed with the eight-item JBI checklist and case series with the ten-item JBI checklist. No numerical summary score or exclusion threshold was applied. CARE domains were considered descriptively in the manuscript without assigning summary reporting categories

### Data Synthesis and Sensitivity Analyses

The substantial clinical and methodological heterogeneity of the included publications precluded meta-analysis. Findings were therefore synthesized qualitatively and summarized using descriptive patient-level reporting, with reporting informed by the Synthesis Without Meta-analysis guideline [19]. Results were expressed as reporting frequencies among published patients and were not interpreted as estimates of prevalence, incidence, absolute risk, or prognosis. Denominators reflected the number of patients with explicitly reported or classifiable information for each variable. Unknown or insufficiently reported findings were retained as missing information and were not interpreted as negative findings. The primary synthesis included all adjudicated food-associated cases, while separate mechanistic categories were applied to confirmed or clinically suspected food allergy, scombroid poisoning caused by exogenous histamine, and other non-immune food-related toxic reactions.

Sensitivity analyses were planned by restricting the synthesis to the confirmed or suspected food-allergy stratum, restricting it to patients classified as having definite or probable KS, and excluding patients in whom epinephrine preceded the onset or first objective documentation of coronary manifestations. The variables included in these analyses were sex, acute electrocardiographic abnormalities, troponin elevation, fixed obstructive coronary lesions or thrombosis, and KS phenotype. For electrocardiographic abnormalities, troponin elevation, and fixed obstructive coronary lesions or thrombosis, effective denominators were based on the number of patients with known or classifiable status within each analytical stratum. The sensitivity analyses were descriptive, and no inferential comparisons were performed. The complete findings are presented in Table 4.

## Results

### Study Selection and Characteristics of the Included Evidence

The database searches identified 2,965 records, including 909 from PubMed, 1,085 from Scopus, and 971 from Web of Science Core Collection. After removal of 1,557 duplicates, 1,408 unique database records underwent title and abstract screening. Of these, 1,318 records were excluded and 90 reports were sought for full-text retrieval. Eight reports could not be retrieved, leaving 82 reports for eligibility assessment. Fifty reports were excluded after full-text review: 14 were not primary research or were review articles, 12 were not food-related or did not meet the criteria for Kounis syndrome, nine lacked objective coronary involvement, seven provided insufficient patient-level information, five were duplicate reports or described an already included patient, and three were excluded for other reasons. Thirty-two eligible publications were retained from the database search. An additional 18 eligible publications were identified through supplementary methods, including eight through backward reference checking, six through forward citation searching, and four through targeted web searching. The final systematic qualitative synthesis included 50 eligible publications or reports describing 53 unique patients. The complete study-selection process is shown in Fig. 1.

The systematic qualitative synthesis included 50 eligible publications or reports describing 53 unique patients with food-associated KS. Multiple publications describing the same patient were linked and counted only once, whereas independently identifiable patients reported within case series were analyzed separately. The clinical index case was analyzed separately and was not included in these totals. Four potentially relevant conference reports underwent full-text or full-report assessment. The abstract by Arends was retained because it provided sufficient patient-level information for inclusion, whereas three reports were excluded because the available information was insufficient for reliable data extraction or assessment of potential patient overlap. Two database records referring to the same conference communication on scombroid poisoning were treated as duplicates.

Patient-level demographic, exposure-related, clinical, and cardiovascular characteristics are presented in Table 1A, whereas laboratory, allergological, treatment, and outcome findings are presented in Table 2B. Aggregate findings are summarized in Table 3, and the corresponding sensitivity analyses are reported in Table 4. The published evidence predominantly involved male and middle-aged or older patients, although the reported age range extended from adolescence to advanced age. Cardiovascular backgrounds ranged from no documented coronary disease to established atherosclerosis, previous myocardial infarction, ischemic cardiomyopathy, and previous coronary stent or bypass-graft implantation. Two patients had underlying clonal or nonclonal mast-cell activation disorders [20].

Reported food-associated exposures were heterogeneous and included fish and shellfish, wheat-containing foods, nuts and peanuts, fruits, vegetables, dairy products, mushrooms, lentils, a branched-chain amino acid supplement, and mixed or incompletely characterized food exposures (Tables 1A and 3). In some reports, the individual causal ingredient could not be established because multiple foods were consumed simultaneously, a concomitant medication represented a competing exposure, or sufficient allergological confirmation was unavailable [21–23]. Food-related mechanisms were assigned to mutually exclusive categories. The confirmed or clinically suspected food-allergy stratum included 46 patients, comprising 20 patients with confirmed food allergy and 26 with clinically suspected food allergy; IgE mediation was demonstrated only when supported by specific allergological findings. Six patients had scombroid poisoning attributed to exogenous histamine [24–29], whereas one patient had another non-immune toxic reaction associated with the interaction between Coprinopsis atramentaria and alcohol [30]. These non-immune presentations were retained in the broad synthesis of food-associated cases because of their clinical relevance but were analyzed separately from the food-allergy stratum.

Potential cofactors and contributing exposures included physical exercise, aspirin or other nonsteroidal anti-inflammatory drugs, alcohol, concomitant medication, recurrent exposure, underlying mast-cell disorders, previous coronary stent implantation, and epinephrine administered during management of the hypersensitivity reaction. Exercise was reported in mango- and wheat-associated presentations [31–34], whereas aspirin or another nonsteroidal anti-inflammatory drug was considered a possible contributing exposure in selected cases [31, 32]. These exposures were recorded descriptively and were not regarded as establishing a causal cofactor relationship.

### Clinical, Cardiovascular, and Allergological Findings

Clinical manifestations varied substantially among the published patients and included cutaneous or mucosal, respiratory, gastrointestinal, and hemodynamic manifestations. Chest pain was commonly reported but was not universal, and cardiac involvement was identified in some patients through electrocardiography, cardiac troponin measurement, echocardiography, cardiac magnetic resonance imaging, or coronary imaging despite the absence of typical chest discomfort. Severe presentations included ventricular fibrillation, profound hemodynamic instability, native-coronary thrombosis, acute stent thrombosis, and thrombosis involving a previously stented saphenous vein graft [23, 34–41]. Cardiopulmonary resuscitation was reported in two patients [35, 39]. Patient-level clinical manifestations and cardiovascular findings are presented in Table 1A.

Latency was retained using the terminology reported in the original publications. Qualitative descriptions such as “shortly after ingestion,” “rapidly,” and “after ingestion” were not converted into quantitative intervals. Consequently, no dichotomous classification based on onset within or after 1 h was applied when the original report did not provide a sufficiently precise time estimate. The temporal relationship between the allergic and cardiovascular manifestations was also heterogeneous, with coronary findings occurring concurrently with the hypersensitivity reaction, after initial treatment, after apparent improvement of the allergic manifestations, or during a biphasic clinical course (Table 1A).

Acute ECG status was classifiable in most patients, and abnormalities included ST-segment elevation or depression, ischemic T-wave changes, atrioventricular block, ventricular bigeminy, ventricular fibrillation, and other dynamic ischemic patterns. Clemen et al. described troponin elevation and a regional wall-motion abnormality without acute ST-segment changes [42], whereas Monerris-Aguilar et al. reported a normal ECG despite troponin elevation, regional hypokinesia, and underlying coronary disease [43]. Aggregate ECG and troponin findings are summarized in Table 3, with variable-specific denominators based on patients with known status. Troponin status was considered classifiable when a numerical value and reference limit were reported or when the authors explicitly documented biomarker-confirmed myocardial injury or myocardial infarction. Direct comparison of troponin concentrations was not undertaken because the publications used different troponin I and troponin T assays, reference intervals, units, and sampling times. Troponin elevation alone was not considered sufficient to establish KS without additional evidence supporting acute myocardial ischemia.

Coronary assessment ranged from invasive coronary angiography and coronary CT angiography to optical coherence tomography and other intracoronary imaging techniques. The reported findings included normal or nonobstructive coronary arteries, inducible or nitrate-responsive vasospasm, fixed stenosis, plaque-related myocardial infarction, native-coronary thrombosis, and stent or graft thrombosis. Patient-level coronary findings are presented in Table 1A, while aggregate reporting frequencies are provided in Table 3. Direct angiographic or pharmacological demonstration of coronary vasospasm was documented in a minority of patients, including cases associated with Pleurotus ostreatus, mango, shellfish-containing noodles, raw fish gallbladder, ice cream, and scombroid poisoning [25, 31, 44–47]. In other type I presentations, a transient spastic mechanism was inferred from the clinical chronology, transient ischemic abnormalities, and absence of fixed obstructive coronary disease. Normal coronary angiography was not considered to exclude transient vasospasm, particularly when coronary assessment occurred after antiallergic treatment or administration of nitrates or other vasodilators.

Type I was the most commonly assigned KS phenotype among published patients, followed by types II and III, while a small number of patients remained unclassifiable because the available cardiovascular information was insufficient. Author-reported phenotypes were retained when supported by the published coronary findings. Reviewer-derived phenotypes were assigned when the original authors did not provide a classification or when interpretation of the reported coronary anatomy and cardiovascular findings was required. The case reported by Jariwala et al. was retained as type III, although the authors also discussed compatibility with a proposed type IV phenotype involving a coronary bypass graft [36]. The first patient reported by Chardin et al. was retained as author-reported type I, but reviewer adjudication remained uncertain because right coronary artery occlusion was also reported [34]. Patient-level phenotype attribution and diagnostic certainty are presented in Table 1A, and their aggregate distributions are summarized in Table 3.

KS diagnostic certainty was evaluated independently of phenotype, food causality, and presumed biological mechanism. Cases were classified as definite, probable, or possible according to the completeness and coherence of the hypersensitivity chronology, objective evidence of myocardial ischemia, coronary findings, and plausible alternative explanations. This distinction allowed a patient to have well-supported coronary involvement but uncertain attribution to a particular food, as occurred in reports involving mixed food ingestion, an unresolved culprit ingredient, or concomitant medication exposure [21–23].

Allergological and mast-cell assessments were inconsistently reported. Skin prick or prick-to-prick testing, food-specific IgE, component-resolved diagnostics, oral food challenge or documented food re-exposure, and serum tryptase were evaluated separately. Positive testing was interpreted as evidence of sensitization in the context of the clinical history and not as independent proof that the sensitized food caused the coronary event. Conversely, absent or negative allergological testing did not automatically exclude a clinically compatible food-associated reaction. Clearly identifiable paired acute and baseline serum tryptase measurements were available in four patients [20, 30, 34, 48]. Molecular findings reported in selected patients included sensitization to ω−5 gliadin, nonspecific lipid transfer proteins, and Sin a 2 [33, 34, 38, 41, 43, 49]. A supervised sheep-milk challenge reproduced anaphylaxis without myocardial injury in the patient reported by Tzanis et al. [37]. Overall reporting frequencies for allergological and mast-cell assessments are presented in Table 3, and the corresponding patient-level findings are provided in Table 2B.

### Epinephrine, Treatment, Outcomes, and Sensitivity Analyses

Epinephrine chronology was assessed descriptively and was not interpreted as evidence of causality. Epinephrine was explicitly administered in 20 of 53 patients (37.7%). It preceded the onset or first objective documentation of coronary manifestations in 12 patients (22.6%) [22, 29, 30, 38–40, 42, 43, 50–52, 63]. Toya et al. and Lin et al. were considered separately because epinephrine preceded a reported ischemic worsening but did not precede the initial objective coronary manifestations [31, 47]. These patients were therefore counted among those who received epinephrine but were not classified as epinephrine-first cases. In Mejía-Rentería et al., Fiore et al., and Chardin et al. patient 1, coronary manifestations or their first objective documentation preceded epinephrine administration [34, 53, 54]. The complete patient-level chronology is presented in Table 2B, and the aggregate findings are summarized in Tables 3 and 5.

Treatment reporting was incomplete and heterogeneous. Treatments were coded only when a specific intervention could be identified explicitly, and NR was not interpreted as absence. Reported cardiovascular management included an explicitly identifiable cardiovascular treatment, intervention, or support in 32 of 53 patients (60.4%). PCI or another therapeutic coronary intervention was reported in 15 patients (28.3%), thrombus aspiration in five (9.4%), coronary vasodilator treatment in 11 (20.8%), and cardiopulmonary resuscitation in two (3.8%). Because these categories were not mutually exclusive, individual patients could contribute to more than one treatment category. Treatment frequencies should therefore be interpreted as frequencies of explicitly reported interventions rather than estimates of treatment utilization. Patient-level treatment and outcome information is presented in Table 2B and summarized in Table 5.

No fatal outcomes were reported in the included publications. This observation should not be interpreted as an estimate of survival or prognosis because of publication bias, selective outcome reporting, and incomplete follow-up. Follow-up of at least 3 months was explicitly documented in six patients (11.3%) [24, 31, 34, 44, 53, 56]. Most publications described acute recovery, hospital discharge, or resolution of cardiovascular abnormalities without standardized long-term allergological or cardiovascular follow-up.

The principal findings remained broadly consistent across the three planned sensitivity analyses. Restricting the synthesis to the confirmed or suspected food-allergy stratum reduced the influence of non-immune food-related reactions; restricting it to definite or probable KS reduced the contribution of cases with limited diagnostic evidence; and excluding epinephrine-first cases addressed uncertainty concerning treatment chronology. These restrictions did not materially alter the overall distributions of KS phenotypes or the reported patterns of ECG abnormalities, troponin elevation, and fixed obstructive coronary disease or thrombosis. Complete results and variable-specific denominators are presented in Table 4. The analyses were descriptive, and no inferential comparisons were performed.

### Methodological Appraisal and Reporting Completeness

Methodological appraisal was conducted using the design-specific JBI Critical Appraisal Checklists for Case Reports and Case Series [16, 17]. Reporting completeness was considered separately and descriptively with reference to relevant CARE domains [18]. No numerical JBI summary score, quality threshold, or high-, moderate-, or low-quality classification was applied, and no publication was excluded on the basis of the appraisal.

The JBI appraisal identified recurrent limitations related to incomplete clinical and treatment timelines, limited evaluation of alternative explanations, inconsistent reporting of adverse or unanticipated events, and incomplete follow-up in case reports. In case series, information concerning inclusion criteria, consecutive or complete recruitment, clinical setting, and patient-identification procedures was frequently unclear. Descriptive consideration of CARE domains similarly identified inconsistent reporting of treatment rationale, adverse events, patient perspective, and long-term follow-up. The conference abstract provided the least complete information because several reporting domains could not be evaluated from an abstract-only source. JBI judgments were interpreted as indicators of methodological strengths and limitations within each study design; the CARE framework was used only to guide descriptive assessment of reporting completeness and not to create summary categories. The complete publication-level JBI assessments are presented in Table 6.

## Discussion

Food-associated Kounis syndrome should be understood as a heterogeneous spectrum of hypersensitivity-associated coronary disease rather than as a uniform clinical entity. The published cases encompassed transient coronary vasospasm, plaque-related myocardial infarction, native-coronary thrombosis, and thrombosis involving coronary stents or previously stented bypass grafts (Tables 1A and 3). This spectrum supports the concept that the coronary phenotype results from the interaction between the systemic hypersensitivity response, the underlying coronary substrate, cardiovascular risk factors, intracoronary devices, and relevant cofactors, rather than from the suspected food alone [1, 4–6]. Although the published evidence predominantly involved middle-aged or older adults, food-associated KS was also described in adolescents and young adults without known structural coronary disease (Table 1A). Pre-existing coronary atherosclerosis is therefore not required for KS to occur. In patients without fixed obstructive disease, mast-cell mediators may predominantly induce epicardial or microvascular spasm. In those with underlying atherosclerosis, previous coronary revascularization, or intracoronary devices, the same inflammatory and prothrombotic response may facilitate plaque destabilization, native-coronary thrombosis, or stent-related complications. Cardiovascular history and coronary anatomy should consequently inform phenotype interpretation but should not determine whether KS is considered.

Type I was the most frequently assigned phenotype, although this finding should be interpreted cautiously (Table 3). Direct angiographic or pharmacological demonstration of coronary vasospasm was documented only in a minority of patients, including cases associated with Pleurotus ostreatus, mango, shellfish-containing noodles, raw fish gallbladder, ice cream, and scombroid poisoning [25, 31, 44–47]. In other type I presentations, a transient spastic mechanism was inferred from reversible ischemic abnormalities, the absence of fixed obstructive coronary disease, and the overall clinical course. Normal coronary angiography does not exclude transient vasospasm, particularly when angiography is performed after symptom improvement or administration of nitrates or other vasodilators. Conversely, apparently normal or nonobstructive angiography may not exclude plaque erosion, transient thrombosis, microvascular dysfunction, or abnormalities detectable only by intracoronary imaging. Gargiulo et al. identified a nonocclusive right coronary thrombus using optical coherence tomography, illustrating the additional mechanistic information that intracoronary imaging may provide [28]. Limitations in the timing and sensitivity of coronary assessment may therefore result in both overestimation and underestimation of type I KS and may explain why some patients remained unclassifiable.

Distinguishing between author-reported and reviewer-derived phenotypes was important because several publications did not provide a classification or contained findings that required further interpretation. The case reported by Jariwala et al. was retained as type III because thrombosis involved a previously stented saphenous vein graft, although the authors also discussed compatibility with a proposed type IV phenotype involving coronary bypass grafts [36]. The first patient reported by Chardin et al. was retained as author-reported type I, but reviewer adjudication remained uncertain because right coronary artery occlusion was also described [34]. These cases highlight the value of presenting the authors’ original classification separately from any reviewer-derived interpretation.

Diagnosis may be particularly challenging when typical chest pain or visible cutaneous involvement is absent. Wada et al. described transient inferior ST-segment elevation and atrioventricular block without chest pain, whereas Tan et al. reported dynamic ischemic abnormalities, inferolateral hypokinesis, and critical right coronary artery stenosis despite the absence of chest pain [46, 56]. More broadly, cardiac involvement was sometimes identified through electrocardiography, cardiac biomarkers, ventricular imaging, or coronary assessment in patients whose initial presentation was dominated by hypotension, syncope, respiratory symptoms, gastrointestinal manifestations, or other features of systemic hypersensitivity (Table 1A). The prominence of cutaneous manifestations in published reports may partly reflect recognition bias, because coronary events are more likely to be attributed to hypersensitivity when the allergic phenotype is clinically apparent.

Prospective evidence also supports the possibility of initially unrecognized myocardial involvement during acute allergic reactions. Anastasopoulou et al. identified myocardial injury through serial electrocardiography and high-sensitivity cardiac troponin measurements, although not every patient with biomarker elevation fulfilled the diagnostic criteria applied for KS [10]. Troponin elevation during an allergic reaction should therefore prompt appropriate cardiovascular assessment but does not independently establish KS. Objective evidence supporting myocardial ischemia, obtained through symptoms, serial electrocardiography, ventricular imaging, coronary assessment, or other relevant investigations, remains necessary. In the same prospective study, myocardial injury was associated with cardiovascular risk factors, cardiovascular comorbidity, severe allergic reactions, syncope, anaphylactic shock, and epinephrine administration [10]. These observations support a low threshold for serial electrocardiography and troponin measurement in patients with severe systemic manifestations, hypotension, syncope, persistent tachycardia, arrhythmia, cardiovascular disease, or symptoms suggestive of myocardial ischemia. The available evidence does not support routine cardiac testing in every mild allergic reaction, but targeted assessment may identify clinically relevant involvement that would otherwise remain unrecognized.

The mechanistic heterogeneity of food-associated presentations requires particular emphasis. Most patients were classified within the confirmed or suspected food-allergy stratum, although IgE mediation was demonstrated only in a subset. Scombroid poisoning caused by exogenous histamine and the non-immune interaction between Coprinopsis atramentaria and alcohol were analyzed separately from conventional food allergy (Table 1A and 3). Scombroid poisoning may reproduce the clinical manifestations of anaphylaxis and may be accompanied by coronary vasospasm, myocardial injury, or thrombosis without previous sensitization to a fish allergen. These presentations are clinically relevant to the differential diagnosis of food-associated coronary events but should not be presented as evidence of IgE-mediated food allergy. The descriptive sensitivity analyses indicate that the principal coronary patterns were not attributable exclusively to these non-immune presentations (Table 4).

Causal interpretation also remained difficult in several patients classified within the food-allergy stratum. Some reports involved mixed meals, an unidentified culprit ingredient, or concomitant medication that represented a competing exposure or potential cofactor [21–23]. Vaina et al. reported an event after ingestion of a green salad without identification of the responsible ingredient, whereas Gomez-Olarte et al. described exposure to tilapia, papaya, and borojó together with amoxicillin-clavulanate [21, 23]. In the patient reported by Hamideh et al., the food exposure and the allergic manifestations preceding epinephrine were incompletely documented [22]. The term food-associated KS is therefore preferable when the temporal association is plausible but the individual food or immunological mechanism cannot be established reliably.

A structured allergological assessment is necessary to distinguish food allergy from toxic reactions, coincidental sensitization, competing medication exposure, and cofactor-dependent hypersensitivity. Positive skin testing, food-specific IgE, or component-resolved diagnostics demonstrate sensitization but do not independently prove that the sensitized food caused the coronary event. Conversely, negative testing does not exclude food allergy, particularly when commercial extracts or available assays do not adequately represent the relevant allergen. This limitation was illustrated by the discordance between allergological tests in the gilthead-fish and lentil cases reported by González-de-Olano et al. [20]. Component-resolved diagnostics may be especially informative when wheat-dependent exercise-induced anaphylaxis, nonspecific lipid transfer protein sensitization, or mustard allergy is suspected. Molecular sensitization to ω−5 gliadin, nonspecific lipid transfer proteins, and Sin a 2 was documented in selected patients (Table 1A). However, detailed molecular testing was reported inconsistently, and the available evidence does not demonstrate a reproducible association between food-specific IgE concentrations, total IgE, serum tryptase, and the severity of coronary involvement. The underlying coronary substrate, the magnitude and timing of mediator release, the presence of cofactors, and the timing of cardiovascular assessment may be more important determinants of the observed phenotype.

Controlled oral exposure or clearly documented food re-exposure was uncommon (Tables 2B and 3). A supervised sheep-milk challenge reproduced anaphylaxis without myocardial injury [37], illustrating that recurrence of the allergic reaction does not necessarily reproduce the same cardiovascular phenotype. The scarcity of controlled or reproducible exposure data substantially limits causal attribution and is understandable given the potential risk of deliberate re-exposure after a severe reaction involving myocardial ischemia.

The present index case expands the limited literature on wheat-associated KS. Published cases indicate that wheat-associated reactions may produce distinct coronary phenotypes. Yaegashi et al. described thrombotic occlusion of the left anterior descending coronary artery during wheat-dependent exercise-induced anaphylaxis, consistent with type II KS [32]. Rosier et al. reported transient myocardial ischemia without obstructive coronary disease, supporting a type I presentation [33]. Watanabe et al. described type III KS with acute stent thrombosis after wheat-bread ingestion in a patient sensitized to ω−5 gliadin [38]. Chardin et al. subsequently reported additional wheat-associated presentations with differing cardiovascular findings [34]. Collectively, these observations reinforce that the allergen itself does not independently determine the coronary phenotype. In the index patient, the temporal relationship between wheat ingestion, ibuprofen exposure, systemic hypersensitivity, and the subsequent coronary event, together with marked sensitization to ω−5 gliadin, supported wheat allergy with ibuprofen as a probable cofactor. The new thrombotic lesion in the left circumflex coronary artery in a patient with established atherosclerotic disease supported a type II phenotype, whereas the patency of the previously implanted stents argued against type III KS. Subsequent tolerance of ibuprofen in isolation supported its interpretation as a cofactor rather than the primary allergenic trigger. Tolerance of gluten-containing food in the absence of relevant cofactors similarly supported a cofactor-dependent threshold rather than clinical reactivity to every wheat exposure.

This distinction has practical consequences. A medication taken close to an allergic reaction may be incorrectly labeled as the primary trigger, resulting in unnecessary long-term avoidance. Conversely, complete elimination of a food may be recommended even when reactions occur only in the presence of a relevant cofactor. Detailed reconstruction of food intake, medication exposure, physical activity, alcohol consumption, previous tolerance, sensitization profile, and symptom chronology can substantially modify the final causal assessment and management recommendations. Any planned challenge or re-exposure after severe KS requires individualized risk assessment, informed consent, appropriate cardiovascular monitoring, and close collaboration between allergology and cardiology.

Physical exercise and nonsteroidal anti-inflammatory drugs were among the principal potential cofactors identified in the published cases (Table 1A). Exercise was reported in mango- and wheat-associated presentations [31–34], whereas aspirin or another nonsteroidal anti-inflammatory drug was considered a possible contributing exposure in selected cases [31, 32]. Exercise may increase intestinal permeability and allergen absorption, while nonsteroidal anti-inflammatory drugs may modify gastrointestinal allergen uptake and arachidonic acid metabolism, thereby reducing the reaction threshold. Alcohol may exert analogous effects but was reported less consistently. Incomplete documentation of cofactors may lead to misclassification of the causal food, the role of concomitant medication, and the underlying reaction mechanism.

Serum tryptase may support systemic mast-cell activation, but its interpretation depends on the sampling interval and comparison with a subsequent baseline concentration. Paired acute and baseline measurements were uncommon in the published evidence (Tables 2B and 3). A normal acute tryptase concentration does not exclude food-associated anaphylaxis, whereas a single elevated value cannot reliably distinguish acute activation from a persistently elevated baseline associated with hereditary α-tryptasemia or a clonal mast-cell disorder. Paired measurements are therefore more informative than isolated determinations. In the index patient, basal tryptase concentrations were repeatedly normal and stable, and peripheral blood testing did not detect the KIT D816V mutation. These findings did not support an underlying clonal mast-cell disorder. TPSAB1 copy-number analysis was not performed because basal tryptase remained below 8 µg/L. Although a normal basal tryptase concentration makes a persistently elevated basal tryptase phenotype less likely, it does not independently exclude hereditary α-tryptasemia. These findings should not be interpreted as supporting or refuting the causal role of wheat in the index event.

Comparison with drug-associated KS is clinically informative but should remain cautious. Drugs, particularly antibiotics and nonsteroidal anti-inflammatory drugs, are among the most frequently reported KS triggers, although contrast media, anesthetic agents, and antineoplastic drugs have also been implicated [1, 5, 7, 58]. The broadly similar range of coronary phenotypes reported across trigger categories supports the hypothesis that the underlying coronary substrate and systemic hypersensitivity response may be more important determinants of phenotype than the trigger category itself. Food and drug exposures nevertheless occur in different clinical contexts. Parenteral medications can produce abrupt systemic exposure, whereas ingested food allergens undergo digestion and absorption and may depend on exercise, nonsteroidal anti-inflammatory drugs, alcohol, or other cofactors to exceed the reaction threshold. Drug-associated events may also be recognized more readily because they frequently occur in monitored healthcare settings. Current evidence does not establish that drug-associated KS is intrinsically more severe, more rapid, or more thrombotic than food-associated KS. Comparisons between trigger categories should therefore remain descriptive.

Management is challenging because the hypersensitivity and coronary components must be treated simultaneously. Intramuscular epinephrine remains the first-line treatment for anaphylaxis and should not be withheld solely because KS is suspected [5, 13, 14]. Its adrenergic effects may nevertheless aggravate coronary vasospasm, increase myocardial oxygen demand, or precipitate arrhythmia in susceptible patients. Clinical decisions must therefore balance the immediate risk of untreated anaphylaxis against the patient’s cardiovascular status. The chronology of epinephrine administration was treated as a potential source of confounding rather than evidence of causality (Tables 2B and 5). Toya et al. and Lin et al. were considered separately because epinephrine preceded a reported worsening of ischemic manifestations but did not precede the initial objective coronary manifestations [31, 47]. More generally, patients receiving epinephrine tended to have more severe hypersensitivity reactions, creating substantial confounding by indication. The association between epinephrine administration and myocardial injury reported by Anastasopoulou et al. cannot distinguish a pharmacological effect from the severity of the underlying reaction [10]. The recommended intramuscular dose and route should therefore be maintained, while intravenous boluses should be reserved for refractory anaphylaxis managed under continuous cardiovascular monitoring [5, 13, 14]. Exclusion of epinephrine-first cases did not materially change the overall distribution of coronary phenotypes (Table 4). This finding does not demonstrate the cardiovascular safety of epinephrine and should not be interpreted causally. It indicates only that the principal pattern observed in the synthesis was not confined to patients in whom epinephrine preceded the coronary manifestations.

Cardiovascular management should be guided by coronary phenotype and hemodynamic status. Nitrates and calcium-channel blockers may be considered in vasospastic presentations when blood pressure permits. Plaque-related myocardial infarction, native-coronary thrombosis, and stent or graft thrombosis require treatment based on acute coronary syndrome protocols and the documented coronary anatomy [1, 5, 7, 9]. The interventions reported in the included literature ranged from coronary vasodilator treatment to thrombus aspiration, percutaneous coronary intervention, and cardiopulmonary resuscitation (Table 5). Some conventional cardiovascular treatments may have particular implications in KS. Aspirin and other nonsteroidal anti-inflammatory drugs may aggravate hypersensitivity in susceptible patients, β-blockers may reduce the response to epinephrine and potentially favor unopposed α-adrenergic vasoconstriction, and some opioids can promote non-IgE-mediated mast-cell activation [1, 5, 7, 9, 59]. These concerns are based primarily on pathophysiological reasoning, case-based evidence, and expert recommendations rather than comparative clinical trials. Management should therefore be individualized and multidisciplinary.

Treatment reporting was incomplete and heterogeneous, and an unreported intervention could not be interpreted as absent (Tables 2B and 5). The available evidence consequently does not permit comparative conclusions regarding the effectiveness or safety of individual treatment strategies. Similarly, the absence of reported fatal outcomes should not be interpreted as evidence of a favorable prognosis. Case reports are susceptible to publication bias, selective outcome reporting, and incomplete follow-up. Food-associated KS should not be regarded as a benign complication of allergy, given the documented occurrence of shock, ventricular fibrillation, native-coronary thrombosis, stent or graft thrombosis, and complicated coronary interventions.

Administrative and observational evidence across all KS triggers has suggested greater in-hospital morbidity than in allergic presentations without coronary involvement [60]. However, such findings cannot be extrapolated directly to food-associated cases because of differences in trigger distribution, case definitions, clinical severity, and data sources. The outcomes observed in the present case-based synthesis should therefore be interpreted together with, but not equated to, findings from broader KS populations.

The methodological appraisal reinforces the need for conservative interpretation. Use of design-specific JBI checklists identified recurrent limitations involving incomplete timelines, limited assessment of alternative explanations, inconsistent treatment reporting, unclear documentation of adverse or unanticipated events, incomplete follow-up, and uncertain consecutive or complete inclusion in case series. Descriptive consideration of CARE domains similarly identified variability in reporting completeness, particularly for treatment rationale, adverse events, patient perspective, and long-term outcomes. Methodological appraisal and reporting completeness represent related but distinct constructs, and neither overcomes the fundamental limitations of uncontrolled case-based evidence.

The principal strength of this synthesis is its structured patient-level assessment of food exposure, hypersensitivity manifestations, objective cardiovascular findings, coronary phenotype, diagnostic certainty, food-related mechanism, epinephrine chronology, allergological evaluation, treatment, and outcome. The expanded search across PubMed, Scopus, and Web of Science Core Collection, removal of the previous lower publication-date restriction, and inclusion of food-specific search terms improved coverage and reproducibility. Separate sensitivity analyses also reduced the risk of treating non-immune toxic reactions, diagnostically possible cases, and epinephrine-first presentations as a homogeneous evidence base.

Future multicenter registries should standardize the chronology of food and medication exposures, potential cofactors, allergic and cardiovascular manifestations, epinephrine administration, serial electrocardiography and cardiac biomarkers, acute and baseline tryptase measurements, ventricular and coronary imaging, allergological assessment, coronary phenotype, treatment, adverse events, and long-term outcomes. Prospective data are needed to improve causal attribution, distinguish coronary ischemia from nonischemic myocardial injury, identify patients at greatest risk, and develop evidence-informed diagnostic and therapeutic pathways.

## Study Limitations

This systematic qualitative synthesis is limited by the case-based nature of the available evidence. Most included publications were individual case reports or small case series, which are particularly susceptible to publication bias, selective reporting, and preferential publication of severe, unusual, angiographically striking, or diagnostically complete presentations. The observed reporting frequencies should therefore not be interpreted as estimates of incidence, prevalence, absolute risk, trigger-specific frequency, comparative severity, or prognosis. Reporting of clinically relevant variables was heterogeneous and frequently incomplete. The chronology of food exposure, allergic manifestations, cardiovascular symptoms, epinephrine administration, and other treatments was not consistently described, while serial electrocardiography, troponin kinetics, echocardiography, cardiac magnetic resonance imaging, coronary imaging, and responses to coronary vasodilators were reported unevenly. These limitations reduced the reproducibility of diagnostic adjudication and may have affected KS phenotype classification. Coronary angiography was not performed or could not be confirmed in every patient, and intracoronary imaging was exceptional. Consequently, transient vasospasm, plaque erosion, microvascular dysfunction, and transient coronary thrombosis may have been underrecognized. Normal coronary angiography did not exclude a vasospastic mechanism, particularly when performed after antiallergic or vasodilator treatment, whereas the absence of fixed obstructive disease did not establish vasospasm unless it was directly visualized or reproduced by pharmacological provocation.

Allergological evaluation was neither standardized nor systematically performed. Food-specific IgE, component-resolved diagnostics, skin testing, oral food challenge or documented re-exposure, and paired acute and baseline tryptase measurements were available in only a subset of patients. Positive allergological testing demonstrated sensitization but did not independently establish that the food caused the coronary event, while negative or unavailable testing did not exclude a clinically compatible food-associated reaction. Food-causality assessment was further complicated by mixed meals, unidentified ingredients, concomitant medications, and incompletely reported cofactors. In some publications, a specific causal food could not be distinguished from other components of a mixed exposure or from a competing medication trigger [21–23]. Diagnostic certainty of KS, certainty of food causality, presumed biological mechanism, and KS phenotype were therefore adjudicated as separate dimensions. The broad synthesis also included six patients with scombroid poisoning caused by exogenous histamine [24–29] and one patient with a non-immune toxic reaction associated with the Coprinopsis atramentaria-alcohol interaction [30]. These presentations may clinically resemble anaphylaxis but should not be interpreted as conventional food allergy. They were retained because of their clinical relevance and examined separately in the mechanistic sensitivity analysis.

Epinephrine was explicitly administered in 20 patients, and it preceded the onset or first objective documentation of coronary manifestations in 12 patients [22, 29, 30, 38–40, 42, 43, 50–52, 63]. Toya et al. and Lin et al. were considered separately because epinephrine preceded a reported ischemic worsening but did not precede the initial objective coronary manifestations [31, 47]. In Mejía-Rentería et al., Fiore et al., and Chardin et al. patient 1, coronary manifestations or their first objective documentation preceded epinephrine administration [34, 53, 54]. These temporal relationships introduced a potential source of confounding but did not establish causality. Confounding by indication was likely because epinephrine was generally administered during more severe allergic reactions. Treatment frequencies were based only on interventions that could be explicitly identified in the publications. NR was not interpreted as absence, and the reported frequencies probably underestimate the use of antihistamines, corticosteroids, intravenous fluids, oxygen, antiplatelet agents, anticoagulation, coronary vasodilators, and other supportive treatments. Treatment categories were not mutually exclusive, and the descriptive evidence does not permit comparative conclusions regarding treatment effectiveness or safety. No fatal outcomes were reported, but follow-up was generally limited and inconsistently described. The absence of reported mortality should not be interpreted as an estimate of survival or prognosis because publication and selective-reporting bias may have influenced the available evidence. Although the search covered PubMed, Scopus, and Web of Science Core Collection and was supplemented by backward reference checking, forward citation searching, and targeted web searching, eight potentially relevant reports could not be retrieved, and eligible reports may also have been missed because of incomplete indexing, inconsistent terminology, conference-only dissemination, or insufficient clinical information. The review was not prospectively registered, and no formal protocol was published. Finally, the design-specific JBI appraisal describes methodological limitations, while the descriptive CARE-informed assessment addresses reporting completeness; neither should be interpreted as a measure of causal certainty, treatment effectiveness, generalizability, or overall certainty of the evidence.

## Conclusions

Food-associated Kounis syndrome represents a heterogeneous spectrum of hypersensitivity-associated coronary disease, ranging from transient coronary vasospasm to plaque-related myocardial infarction, native-coronary thrombosis, and stent or coronary graft thrombosis. The food-associated literature also includes clinically similar non-immune presentations caused by exogenous histamine and other food-related toxic mechanisms, which should be distinguished from confirmed or clinically suspected food allergy. KS should be considered when allergic or hypersensitivity manifestations following food ingestion coexist with chest discomfort, dyspnea, syncope, hypotension, arrhythmia, ischemic electrocardiographic abnormalities, myocardial injury, or new ventricular dysfunction. Cardiac involvement may occur without typical chest pain and may become apparent only through serial electrocardiography, cardiac biomarker measurement, ventricular imaging, or coronary assessment. Troponin elevation during an allergic reaction warrants cardiovascular evaluation but does not independently establish KS.

Diagnosis requires integration of the exposure and symptom chronology, objective cardiovascular findings, underlying coronary substrate, cardiovascular imaging, potential cofactors, competing exposures, and timing of treatment. Normal coronary angiography does not exclude transient vasospasm, particularly when performed after antiallergic or vasodilator treatment, and diagnostic certainty, KS phenotype, food causality, and presumed biological mechanism should be assessed separately. A structured post-event allergological evaluation is important to identify the most likely causal food, distinguish food allergy from scombroid poisoning and other toxic reactions, evaluate relevant cofactors, and avoid unnecessary dietary or medication restrictions. Evidence of sensitization must be interpreted in the context of the clinical history, and paired acute and baseline tryptase measurements are preferable to isolated measurements when mast-cell activation is being assessed.

Management requires simultaneous treatment of the allergic and coronary components according to the patient’s hemodynamic status and coronary phenotype. Intramuscular epinephrine remains the first-line treatment for anaphylaxis and should not be withheld solely because KS is suspected. Its timing and route should be documented carefully, but the occurrence of coronary manifestations after epinephrine should not be interpreted as proof of causality. The available evidence remains insufficient to estimate prognosis or compare diagnostic and therapeutic strategies. Prospective multidisciplinary registries using standardized definitions, structured allergological assessment, consistent cardiovascular characterization, detailed treatment chronology, and long-term follow-up are needed to improve phenotyping, causal assessment, and evidence-informed management.

## Summary Boxes

### Points for Clinical Practice

- Food-associated KS should be considered when allergic manifestations following food exposure coexist with chest discomfort, dyspnea, syncope, hypotension, arrhythmia, ischemic electrocardiographic abnormalities, or evidence of myocardial injury.
- Electrocardiography and serial cardiac troponin measurement should be considered in patients with allergic reactions who develop cardiovascular symptoms, syncope, hypotension, severe systemic manifestations, arrhythmia, or relevant cardiovascular risk factors.
- Management should address the allergic and coronary components simultaneously and should be guided by hemodynamic status and coronary phenotype. Intramuscular epinephrine remains the first-line treatment for anaphylaxis, and temporal precedence alone should not be interpreted as evidence of coronary causality.
- Structured post-event allergological evaluation is recommended to distinguish food allergy from non-immune food-related reactions, identify relevant cofactors, and avoid unnecessary dietary or medication restrictions.

### Questions for Future Research

- Which clinical, immunological, and cardiovascular factors predict severe coronary involvement or life-threatening outcomes in food-associated KS?
- Which standardized criteria can best distinguish food-allergic KS from scombroid poisoning, other non-immune food-related toxic reactions, and cardiovascular manifestations occurring after epinephrine administration?
- How do exercise, nonsteroidal anti-inflammatory drugs, alcohol, and other cofactors influence reaction threshold, latency, severity, and coronary phenotype?
- Can prospective multidisciplinary registries improve KS phenotyping, causal attribution, diagnostic accuracy, treatment selection, and long-term outcome assessment?
- What is the optimal post-event allergological evaluation for balancing diagnostic accuracy with cardiovascular safety after suspected food-associated KS?

## Supplementary Material

Below is the link to the electronic supplementary material.

Supplementary Material 1

## Data Availability

Provide a statement that lets us know if you have used data, where to find it, and how to access it. This applies whether your data is newly generated or from existing sources. Sharing data improves transparency and reproducibility. Learn more about it on our Data availability statements page (opens in a new window).The option you select must be consistent with your manuscript file.Have you used or generated research data in this study? No or not applicable. This manuscript does not report data generation or analysis.Yes. I used or generated research data in this study.The statement added here will replace any statement written within the manuscript and is the one that we will publish. If you don’t have one yet, see some example statements that you may adapt (opens in a new window).
