# Supplementary material for: Food-Associated Kounis Syndrome: Clinical Spectrum and Diagnostic Considerations from a Case Report and Systematic Qualitative Synthesis of Published Cases
Source: Clin Rev Allergy Immunol. 2026 Oct 11;69(1):84. doi: 10.1007/s12016-026-09210-1 (PMC13653854; doi:10.1007/s12016-026-09210-1)
Supplement: Supplementary file 1 [file 12016_2026_9210_MOESM1_ESM.docx]

**Supplementary Material**

**Supplementary Table S1. PRISMA 2020 Checklist**

**Manuscript title:** Food-Associated Kounis Syndrome: Clinical Spectrum and Diagnostic Considerations from a Case Report and Systematic Qualitative Synthesis of Published Cases

**Review type:** Systematic qualitative synthesis of case reports and case series without meta-analysis

| **Section and topic** | **Item** | **Checklist item** | **Location where item is reported** |
| --- | --- | --- | --- |
| TITLE | 1 | Identify the report as a systematic review. | Title page |
| ABSTRACT | 2 | See the PRISMA 2020 for Abstracts checklist. | Abstract |
| INTRODUCTION | 3 | Describe the rationale for the review in the context of existing knowledge. | Introduction |
| INTRODUCTION | 4 | Provide an explicit statement of the objectives or questions addressed by the review. | Introduction, final paragraph |
| METHODS | 5 | Specify inclusion and exclusion criteria and how studies were grouped for synthesis. | Methods, Eligibility Criteria; Data Synthesis and Sensitivity Analyses |
| METHODS | 6 | Specify all databases, websites, reference lists, and other sources searched, and the last search date. | Methods, Information Sources and Search Strategy; Supplementary Table S2 |
| METHODS | 7 | Present the complete search strategy for each source, including fields, limits, and filters. | Supplementary Table S2 |
| METHODS | 8 | Specify the process used to decide whether reports met the inclusion criteria. | Methods, Record Management and Study Selection |
| METHODS | 9 | Specify the data-collection process and any procedures used to confirm unclear information. | Methods, Data Extraction |
| METHODS | 10a | List and define all outcomes for which data were sought. | Methods, Data Extraction |
| METHODS | 10b | List and define all other variables and assumptions regarding missing or unclear information. | Methods, Data Extraction; Diagnostic and Causal Adjudication |
| METHODS | 11 | Specify the methods used to assess methodological limitations in included reports. | Methods, Methodological Appraisal and Reporting Completeness |
| METHODS | 12 | Specify the effect measure used for each outcome. | Not applicable; no comparative effect measures were calculated. Findings were summarized as counts and reporting frequencies. |
| METHODS | 13a | Describe the process used to decide which reports were eligible for each synthesis. | Methods, Eligibility Criteria; Diagnostic and Causal Adjudication; Data Synthesis and Sensitivity Analyses |
| METHODS | 13b | Describe methods used to prepare data for presentation or synthesis. | Methods, Data Extraction |
| METHODS | 13c | Describe methods used to tabulate or visually display results. | Methods, Data Synthesis and Sensitivity Analyses; Tables 1A-5; Figure 1 |
| METHODS | 13d | Describe methods used to synthesize results and justify the selected approach. | Methods, Data Synthesis and Sensitivity Analyses; SWiM Compliance Statement |
| METHODS | 13e | Describe methods used to explore possible causes of heterogeneity. | Methods, Diagnostic and Causal Adjudication; Data Synthesis and Sensitivity Analyses; SWiM Compliance Statement |
| METHODS | 13f | Describe sensitivity analyses used to assess robustness. | Methods, Data Synthesis and Sensitivity Analyses; Table 3 |
| METHODS | 14 | Describe methods used to assess bias due to missing results. | No formal statistical assessment was performed because no meta-analysis was conducted; publication and selective-reporting bias are addressed in Study Limitations. |
| METHODS | 15 | Describe methods used to assess certainty or confidence in the body of evidence. | No formal certainty-of-evidence framework was applied; rationale and implications are reported in Methods and Study Limitations. |
| RESULTS | 16a | Describe the results of the search and selection process, ideally using a flow diagram. | Results, Study Selection and Characteristics of the Included Evidence; Figure 1 |
| RESULTS | 16b | Cite reports that appeared potentially eligible but were excluded, and explain the reasons. | Supplementary table |
| RESULTS | 17 | Cite each included report and present its characteristics. | Results; Tables 1A and 1B |
| RESULTS | 18 | Present the methodological appraisal for each included report. | Results, Methodological Appraisal and Reporting Completeness; Table 5 |
| RESULTS | 19 | Present results for all outcomes for each included report. | Tables 1A and 1B |
| RESULTS | 20a | Summarize characteristics and limitations of reports contributing to each synthesis. | Results; Tables 1A, 1B, 2, and 5 |
| RESULTS | 20b | Present results of statistical syntheses. | Not applicable; no meta-analysis or statistical pooling was performed. |
| RESULTS | 20c | Present results of investigations of possible causes of heterogeneity. | Results, Clinical, Cardiovascular, and Allergological Findings; Sensitivity Analyses; Tables 2 and 3 |
| RESULTS | 20d | Present results of all sensitivity analyses. | Results, Epinephrine, Treatment, Outcomes, and Sensitivity Analyses; Table 3 |
| RESULTS | 21 | Present assessments of bias due to missing results. | No formal statistical assessment was performed; publication and selective-reporting bias are discussed in Study Limitations. |
| RESULTS | 22 | Present assessments of certainty or confidence in the body of evidence. | No formal certainty grading was performed; limitations of case-based evidence are discussed in Study Limitations. |
| DISCUSSION | 23a | Provide a general interpretation of results in the context of other evidence. | Discussion |
| DISCUSSION | 23b | Discuss limitations of the evidence included in the review. | Discussion; Study Limitations |
| DISCUSSION | 23c | Discuss limitations of the review processes used. | Study Limitations |
| DISCUSSION | 23d | Discuss implications for practice and future research. | Discussion; Conclusions; Summary Boxes |
| OTHER INFORMATION | 24a | Provide registration information or state that the review was not registered. | Methods or Study Limitations: the review was not prospectively registered. |
| OTHER INFORMATION | 24b | Indicate where the protocol can be accessed or state that no protocol was prepared. | Methods or Study Limitations: no formal review protocol was published. |
| OTHER INFORMATION | 24c | Describe amendments to registration or protocol. | Not applicable; the review was not prospectively registered and no formal protocol was published. |
| OTHER INFORMATION | 25 | Describe financial or nonfinancial support and the role of funders. | Funding Statement |
| OTHER INFORMATION | 26 | Declare competing interests. | Conflict of Interest Statement |
| OTHER INFORMATION | 27 | Report availability of data, code, and other materials. | Data Availability Statement; Supplementary Material |

**Supplementary Table S2. Complete Database-Specific Search Strategies**

The complete database-specific search strategies used in the review are presented below. Searches were conducted on August 22, 2026, without language or publication-date restrictions.

| **Database** | **Platform/interface** | **Date searched** | **Coverage** | **Complete search strategy** | **Filters/limits** | **Records retrieved** |
| --- | --- | --- | --- | --- | --- | --- |
| PubMed | National Library of Medicine | August 22, 2026 | Database inception to August 22, 2026 | (("Kounis syndrome"[Title/Abstract] OR "allergic acute coronary syndrome"[Title/Abstract] OR "allergic coronary syndrome"[Title/Abstract] OR "allergic angina"[Title/Abstract] OR "allergic myocardial infarction"[Title/Abstract]) OR ((anaphylaxis[Title/Abstract] OR allerg*[Title/Abstract] OR hypersensitiv*[Title/Abstract]) AND (coronary[Title/Abstract] OR myocardial[Title/Abstract] OR "acute coronary syndrome"[Title/Abstract] OR vasospasm[Title/Abstract] OR "stent thrombosis"[Title/Abstract]))) AND (food*[Title/Abstract] OR ingest*[Title/Abstract] OR meal*[Title/Abstract] OR fish[Title/Abstract] OR seafood[Title/Abstract] OR shellfish[Title/Abstract] OR tuna[Title/Abstract] OR wheat[Title/Abstract] OR gluten[Title/Abstract] OR gliadin[Title/Abstract] OR peanut*[Title/Abstract] OR nut*[Title/Abstract] OR fruit*[Title/Abstract] OR kiwi*[Title/Abstract] OR mushroom*[Title/Abstract] OR milk[Title/Abstract] OR lentil*[Title/Abstract] OR mustard[Title/Abstract] OR oyster*[Title/Abstract] OR crab[Title/Abstract] OR shrimp[Title/Abstract] OR Anisakis[Title/Abstract] OR scombroid[Title/Abstract] OR "histamine poisoning"[Title/Abstract] OR Coprinopsis[Title/Abstract]) | No language or publication-date limits | 909 |
| Scopus | Elsevier | August 22, 2026 | Database inception to August 22, 2026 | TITLE-ABS-KEY(("Kounis syndrome" OR "allergic acute coronary syndrome" OR "allergic coronary syndrome" OR "allergic angina" OR "allergic myocardial infarction") OR ((anaphylaxis OR allerg* OR hypersensitiv*) AND (coronary OR myocardial OR "acute coronary syndrome" OR vasospasm OR "stent thrombosis"))) AND TITLE-ABS-KEY(food* OR ingest* OR meal* OR fish OR seafood OR shellfish OR tuna OR wheat OR gluten OR gliadin OR peanut* OR nut* OR fruit* OR kiwi* OR mushroom* OR milk OR lentil* OR mustard OR oyster* OR crab OR shrimp OR Anisakis OR scombroid OR "histamine poisoning" OR Coprinopsis) | No language or publication-date limits | 1,085 |
| Web of Science Core Collection | Clarivate | August 22, 2026 | Database inception to August 22, 2026 | TS=(("Kounis syndrome" OR "allergic acute coronary syndrome" OR "allergic coronary syndrome" OR "allergic angina" OR "allergic myocardial infarction") OR ((anaphylaxis OR allerg* OR hypersensitiv*) AND (coronary OR myocardial OR "acute coronary syndrome" OR vasospasm OR "stent thrombosis"))) AND TS=(food* OR ingest* OR meal* OR fish OR seafood OR shellfish OR tuna OR wheat OR gluten OR gliadin OR peanut* OR nut* OR fruit* OR kiwi* OR mushroom* OR milk OR lentil* OR mustard OR oyster* OR crab OR shrimp OR Anisakis OR scombroid OR "histamine poisoning" OR Coprinopsis) | No language or publication-date limits | 971 |

Supplementary searching: Backward reference checking of included publications and relevant reviews, forward citation searching, and targeted web searching were performed to identify recent, non-indexed, or conference reports. Eligible reports identified through these methods were assessed according to the same inclusion criteria as database-derived records. Eighteen additional eligible publications were identified through supplementary searching, including eight through backward reference checking, six through forward citation searching, and four through targeted web searching. These publications were incorporated into the final qualitative synthesis and are represented separately in the PRISMA flow diagram.

**SWiM Compliance Statement**

**Manuscript:** Food-Associated Kounis Syndrome: Clinical Spectrum and Diagnostic Considerations from a Case Report and Systematic Qualitative Synthesis of Published Cases

**Review type:** Systematic qualitative synthesis of case reports and case series (no meta‑analysis)

This statement describes how the review complies with the Synthesis Without Meta‑analysis (SWiM) reporting recommendations.

**1. Grouping of Studies for Synthesis**

Publications were grouped according to clinically relevant domains defined before the synthesis: demographic characteristics, food-associated exposure, food-related mechanism, potential cofactors or contributing exposures, symptom latency, clinical manifestations, electrocardiographic findings, cardiac biomarkers, ventricular and coronary imaging findings, Kounis syndrome phenotype, diagnostic certainty, allergological and mast-cell assessment, treatment, and outcomes. Publications or reports were used as the unit of analysis for study selection and methodological appraisal. Individual patients were used as the unit of analysis for demographic, clinical, cardiovascular, laboratory, allergological, treatment, and outcome variables. When case series included both food- and non-food-associated triggers, only independently identifiable patients with food-associated events were included in the clinical synthesis.

**2. Standardized Metric and Data Presentation**

No comparative effect estimates were calculated because the evidence consisted of case reports and small case series without control groups. Findings were summarized using patient-level counts and proportions, with variable-specific denominators based on available information. Publication-level counts were used for study selection and methodological appraisal. Laboratory values were converted to common units when mathematically valid. Troponin I and troponin T values were not considered directly interchangeable because the original publications used different assays, reference intervals, analytical sensitivities, and sampling times. Qualitative descriptions such as “elevated,” “normal,” or “negative” were retained without numerical imputation.

**3. Synthesis Methods**

A structured qualitative and descriptive synthesis was performed to characterize the clinical spectrum of food-associated Kounis syndrome. Findings were synthesized narratively and presented in patient-level and aggregate tables. Categorical variables were described using counts and percentages. No statistical pooling, effect-size calculation, or meta-analysis was undertaken because of substantial clinical and methodological heterogeneity in food exposures, potential cofactors, patient characteristics, diagnostic evaluation, laboratory assays, coronary findings, treatment, and outcome reporting.

**4. Criteria for Including Studies in Each Synthesis**

All eligible publications were considered for each relevant synthesis domain. Individual patients contributed only to variables for which information was explicitly available. Information not described in the original publication was recorded as “not reported” and was not interpreted as absent. “Not performed” was used only when the publication explicitly stated that an investigation or procedure had not been undertaken. Missing data were not imputed. When the Kounis syndrome phenotype was not reported by the original authors, it was derived by the reviewers from the coronary history, imaging findings, underlying coronary disease, and presence of intracoronary devices. Reviewer-derived classifications were identified explicitly.

**5. Handling of Heterogeneity**

Clinical and methodological heterogeneity was anticipated and explored qualitatively. Variability was examined across food-associated exposures, food-related mechanisms, potential cofactors, symptom latency, the chronology of allergic and cardiovascular manifestations, epinephrine administration, coronary anatomy, Kounis syndrome phenotype and diagnostic certainty, biomarker findings, allergological and mast-cell assessment, treatment, and outcomes. No formal statistical measure of heterogeneity was calculated.

**6. Certainty of Evidence**

Formal certainty-of-evidence grading was not performed because the evidence consisted predominantly of individual case reports and small case series without comparator groups. Interpretability was considered in relation to publication bias, selective reporting, incomplete clinical information, inconsistent allergological assessment, heterogeneous diagnostic criteria, and possible causal or phenotypic misclassification. These limitations are addressed in the Study Limitations section.

**7. Reporting Bias and Sensitivity Analyses**

No formal statistical assessment of reporting bias was performed because no pooled effect estimate was calculated. The likely preferential publication of severe, unusual, diagnostically complete, or angiographically striking cases was considered narratively. Planned descriptive sensitivity analyses were performed by restricting the synthesis to the confirmed or suspected food-allergy stratum, to patients classified as having definite or probable Kounis syndrome, and to patients without epinephrine administration before the onset or first objective documentation of coronary manifestations. These analyses evaluated the robustness of the principal findings and did not include inferential comparisons.

**8. Transparency and Reproducibility**

The information sources, search strategy, eligibility criteria, study-selection process, data-extraction variables, unit-harmonization procedures, phenotype and diagnostic-certainty criteria, and synthesis methods are described in the Materials and Methods section. Tables 1A and 1B provide patient-level clinical data. Table 2 summarizes the principal demographic, mechanistic, cardiovascular, allergological, and phenotypic findings. Table 3 presents the sensitivity analyses. Table 4 summarizes explicitly reported treatments and outcomes. Table 5 presents the design-specific Joanna Briggs Institute methodological appraisal. CARE domains were considered descriptively without summary categories, and no publication was excluded on the basis of the appraisal.
